# ZnO-based multifunctional nanocomposites to inhibit progression and metastasis of melanoma by eliciting antitumor immunity via immunogenic cell death

**DOI:** 10.7150/thno.44920

**Published:** 2020-09-14

**Authors:** Yamin Zhang, Chen Guo, Liping Liu, Jian Xu, Hao Jiang, Danqi Li, Jiajia Lan, Jun Li, Jing Yang, Qiming Tu, Xiaoyan Sun, Mahin Alamgir, Xiang Chen, Guanxin Shen, Jintao Zhu, Juan Tao

**Affiliations:** 1 Department of Dermatology, Union Hospital, Tongji Medical College, Huazhong University of Science and Technology (HUST), Wuhan 430022, China.; 2 Key Laboratory of Material Chemistry for Energy Conversion and Storage, Ministry of Education, School of Chemistry and Chemical Engineering, HUST, Wuhan 430074, China.; 3 Department of Hematology, Union Hospital, Tongji Medical College, HUST, Wuhan 430022, China.; 4 Department of Dermatology, The Central Hospital of Wuhan, Tongji Medical College, HUST, Wuhan 430022, China.; 5 Clinical Laboratory, Union Hospital, Tongji Medical College, HUST, Wuhan 430022, China.; 6 Department of Dermatology, Tongji Hospital, Tongji Medical College, Huazhong University of Science and Technology (HUST), Wuhan 430022, China.; 7 Department of Dermatology, Rutgers-RWJMS, Somerset, New Jersey, USA.; 8 Department of Dermatology, XiangYa Hospital, Central South University, Changsha 410008, China.; 9 Department of Immunology, Tongji Medical College, HUST, Wuhan 430022, China.

**Keywords:** ZnO Nanoparticles, Immunogenic Cell Death, Thermo-chemotherapy, Mesoporous Silica Nanoparticles, Melanoma

## Abstract

**Rationale:** The development of a highly effective and tumor-specific therapeutic strategy, which can act against the primary tumor and also condition the host immune system to eliminate distant tumors, remains a clinical challenge.

**Methods:** Herein, we demonstrate a facile yet versatile ZnO-capping and Doxorubicin (DOX)-loaded multifunctional nanocomposite (AuNP@mSiO_2_@DOX-ZnO) that integrates photothermal properties of gold nanoparticles (NPs), pH-responsive properties and preferential selectivity to tumor cells of ZnO QDs and chemotherapeutic agent into a single NP. The photothermal performance, pH-triggered release and preferential phagocytic ability were assessed. The induced anti-tumor immunity was determined by analyzing immune cell profile in tumor* in vivo* and molecular mechanism were identified by detecting expression of immunogenic cell death (ICD) markers *in vitro*. Moreover, mice models of unilateral and bilateral subcutaneous melanoma and lung metastasis were established to evaluate the antitumor effects.

**Results:** As an efficient drug carrier, ZnO-capped NPs guarantee a high DOX payload and an *in vitro*, efficient release of at pH 5.0. In murine melanoma models, the nanocomposite can significantly inhibit tumor growth for a short period upon low-power laser irradiation. Importantly, ZnO NPs not only demonstrate preferential selectivity for melanoma cells but can also induce ICD. Meanwhile, AuNP@mSiO_2_-based photothermal therapy (PTT) and DOX are directly cytotoxic towards cancer cells and demonstrate an elevated ICD effect. The induced ICD promotes maturation of dendritic cells, further stimulating the infiltration of effector T cells into tumor sites, preventing tumor growth and distant lung metastases.

**Conclusions:** This study highlights the novel mechanism of ZnO-triggered anti-tumor immunity via inducing ICD. Additionally, we shed light on the multifunctionality of nanocomposites in delivering localized skin tumor therapy as well as inhibiting metastatic growth, which holds great promise in clinical applications.

## Introduction

Malignant melanoma is one of the most aggressive cutaneous cancers 1. Conventional monotherapies (*e.g.*, surgery, chemotherapy and radiotherapy) and novel targeted therapies (*e.g.*, vemurafenib, dabrafenib and trametinib) are limited by poor response rates and treatment relapses 1. Combination immunotherapy (*e.g.*, PD-1 and anti-CTLA4 checkpoint inhibitors) has exhibited extremely high rates of treatment-related adverse events, with Grade 3 adverse events noted in up to 54% of cases 2. Therefore, the development of a highly effective, minimally toxic yet tumor-specific therapeutic strategy is of prime importance. Presently, photothermal inorganic nanoparticles (NPs) (*e.g.*, carbon nanotubes, graphene oxide, gold and CuS NPs) mediated thermo-chemotherapy, which simultaneously deliver heat and chemotherapeutic agents to tumors, has shown great promise and has proven to be effective against lung, breast and cervical carcinomas, hepatomas and melanomas 3-8. Among them, mesoporous silica-coated gold NPs (Au@SiO_2_ NPs), a novel platform, enable facile surface modification and photothermal performance, and can also ensure high drug load efficiency 9-12. However, the drugloaded Au@SiO_2_ delivery system still has multiple limitations in practical applications, including (i) premature drug release due to limited pore blocking of Au@SiO_2_ and damage to normal tissues and organs owing to non-specific drug delivery 13, (ii) inability to eliminate tumor cells at sites of distant metastases, and (iii) the need for use of high intensity (up to 48 W/cm^2^) and long exposure times (up to 40 min) in photothermal-based therapies which mainly dependent on a photothermal effect. This makes its application in the clinical treatments of tumors impractical, as it results in damage to normal tissues 14.

To achieve the goal of targeted delivery of therapeutic payloads to tumor sites, a pH-responsive gatekeeping system was selected as the pH value of tumor cells (pH = 6.5) and intracellular endosomes and lysosomes (pH = 4.0-6.0) is distinctly lower than that of blood and normal tissues (pH = 7.4) 15. Several pH-sensitive gatekeepers, including polyelectrolytes, supramolecular, acid-decomposable inorganic materials (*e.g.*, quantum dots, metal and ceramic NPs) and pH-sensitive linkers, have been employed 16-18. Although these nanocarriers shown promising therapeutic efficacy, some of them are hydrolyzed at rather low pH values (~ 3-4) and their use may involve tedious and complex synthesis steps, which limits their applications 19. As a promising gatekeeper, ZnO QDs are stable at pH of 7.4 but rapidly dissolve in an acidic milieu (at pH <5.5). Meanwhile, ZnO QDs are facile to fabricate, cost-effective, biodegradable, display adequate response to acid at pH < 5.5 and preferentially exhibit cytotoxicity against cancer cells, making them an ideal pH-responsive capping agent for Au@SiO_2_-based delivery system 19-21.

Apart from drug delivery, multiple NPs (*e.g.*, CuS, graphene oxide, ICG or carbon nanotubes) have demonstrated the ability to induce a distinct anti-tumor immune response 4,22. Shen et al showed that ZnO NPs played an adjuvant role as they produce immunoregulatory cytokines in glioblastoma models 23. Taking this into consideration, we postulate that the potential immunoadjuvant effect of ZnO QDs might be helpful in reducing the required power and exposure time of Au@SiO_2_-based thermo-chemotherapy for eliminating primary tumor and inhibit distant metastases by enhancing anti-tumor immunity. At present, the mechanism underlying the generation of a ZnO QDs triggered anti-tumor immune response remains unclear.

Herein, we generated DOX-loaded Au@SiO_2_ with ZnO QDs capped through a facile strategy, integrating photothermal properties of gold NPs, pH-responsive and gatekeeping characteristics of ZnO NPs and chemotherapy agent into a single NP. Interestingly, the nanocomposite exhibited a high DOX payload, with controlled drug release under acidic environments and induction of potent tumor-specific immune responses. Moreover, the nanocomposite exhibited excellent *in vivo* antitumor effects and inhibited lung metastasis with a short irradiation time using low power densities (1.0 W/cm^2^ for 40 s). Additionally, we discuss the mechanisms underlying the generation of a systemic tumor-specific immune response through this combination strategy. Thus, we demonstrate the development of a ZnO-triggered combination therapy strategy that has distinct tumor targeting properties, as well the potential to induce antitumor immunity, without detectable toxic side effects, thus holding great promise as a clinically applicable melanoma therapy.

## Methods

### Preparation of AuNP@mSiO_2_-ZnO

**Figure 1:**
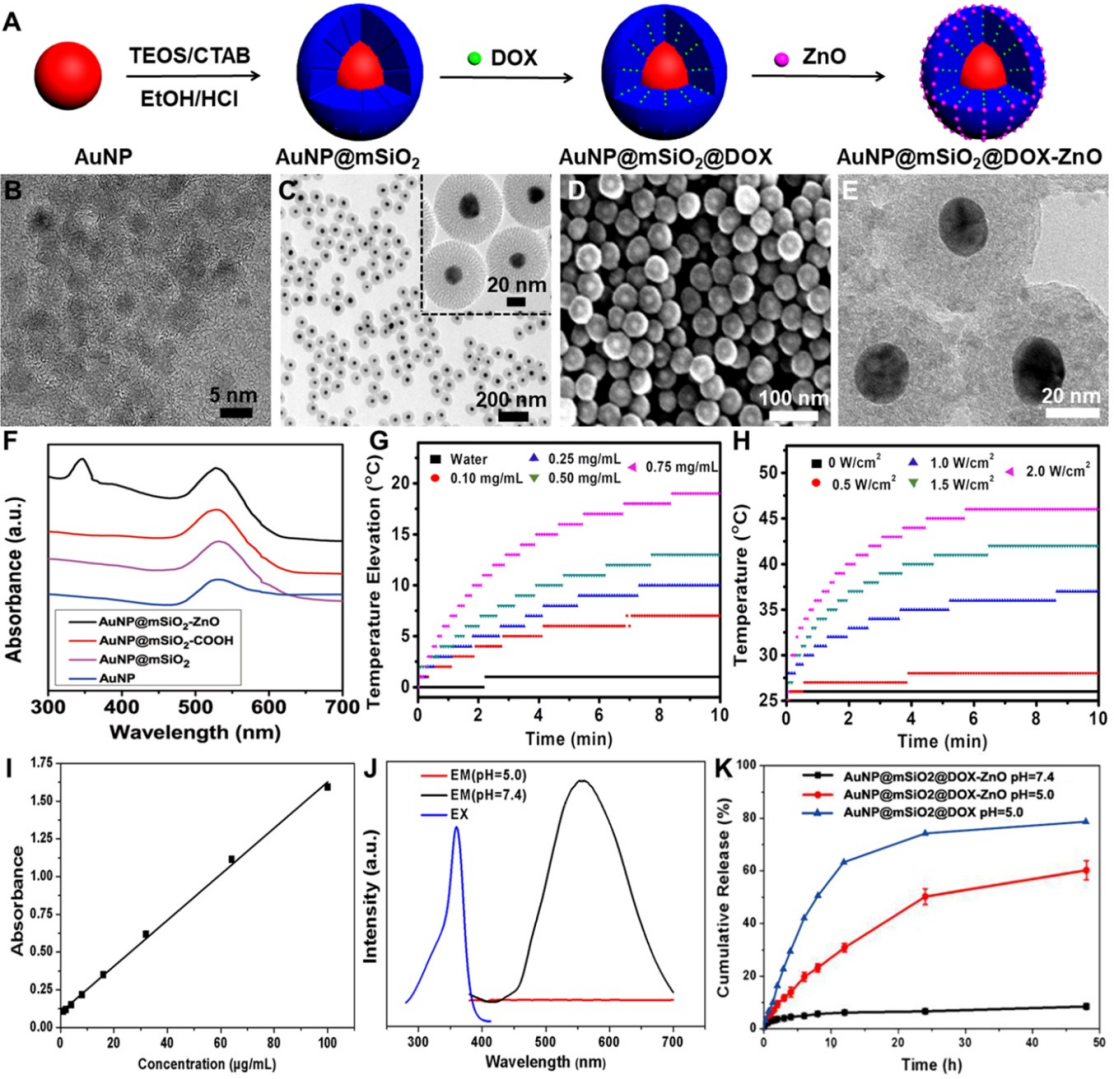
Synthesis, characterization, photothermal properties and controlled release of AuNP@mSiO_2_-ZnO. (A) Preparation procedure of AuNP@mSiO_2_@DOX-ZnO nanocomposites. (B) TEM image of ZnO QDs. (C) TEM image and (D) SEM images of AuNP@mSiO_2_. (E) Electron microscopy image of AuNP@mSiO_2_-ZnO. (F) UV-Vis absorption spectra of AuNP@mSiO_2_-ZnO. (G) The temperature change of aqueous solution containing the AuNP@mSiO_2_-ZnO nanocomposites with different concentrations under 655 nm laser irradiation (2.0 W/cm^2^). (H) The temperature variation of AuNP@mSiO_2_-ZnO solution (0.75 mg/mL) under various power densities of laser. (I) Standard curve of DOX detected with a UV-vis detector. (J) Fluorescence spectroscopy of AuNP@mSiO_2_-ZnO in PBS (pH 7.4) and acetate solution (pH 5.0). EX: Excitation; EM: Emission. (K) The cumulative release kinetics of AuNP@mSiO_2_@DOX-ZnO nanocomposites in PBS (pH 7.4) and acetate solution (pH 5.0) *in vitro* at 37 °C.

AuNP@mSiO_2_-ZnO nanocomposites were prepared through a template method as shown in Figure 1A. Firstly, AuNP@mSiO_2_ NPs were synthesized following a previously reported method 24. Typically, 48 mL of cetyl trimethyl ammonium bromide (CTAB, 0.10 g) aqueous solution were added to a 100 mL flask containing 1.2 mL of 0.5 M NaOH solution. The solution was heated to 80 °C for 15 min with vigorous magnetic stirring, and then 2.0 mL of formaldehyde solution (3.7 wt%) was added, followed by the addition of 1.6 mL of HAuCl_4_ (50 mM) aqueous solution. After 10 min, 1.8 mL of TEOS (20 wt %) solution in ethanol was quickly injected into the above heated solution, and the mixture was heated for another 1 h. After the solution was cooled down to room temperature, the products were centrifuged. The precipitate was redispersed in water and collected by centrifugation. AuNP@mSiO_2_ NPs were obtained after removal of the CTAB micelles in the mesoporous shell by an ion-exchange method. The final AuNP@mSiO_2_-COOH NPs were then functionalized with carboxyl groups by grafting TESPA.

Then, APTES-modified ZnO QDs (ZnO-NH_2_) were synthesized. Briefly, 10 mL of ethanol was added into a 100 mL round-bottom flask, followed by the addition of 10 mL of zincacetate dihydrate (1.86 mM) solution in ethanol. The solution was then refluxed under vigorous magnetic stirring for 2 h in a water bath. Subsequently, 5 mL of KOH (4.11 mM) solution in ethanol was added dropwise. Then, the mixture was cooled down to room temperature and stirred for 1 h to obtain the ZnO QDs. For modification of ZnO QDs, 5 mL of APTES (0.34 mM) solution in ethanol was added to above ZnO QDs suspension. The mixture was stirred for 2 h. ZnO QDs were purified by centrifugation (10000 rpm, 10 min) and washed with deionized water several times to remove excess APTES. The obtained ZnO-NH_2_ was redispersed in deionized water and it emitted yellow fluorescence when irradiated with a 365 nm UV lamp. To prepare AuNP@mSiO_2_-ZnO nanocomposites, AuNP@mSiO_2_-COOH (10 mg) was dispersed in water after which aqueous solution of EDC, NHS and 1.0 mL of ZnO-NH_2_ were added. The mixture was stirred for another 24 h. Afterwards the precipitate was centrifuged, washed several times with water (pH 7.0) and the AuNP@mSiO_2_-ZnO nanocomposites were obtained and dispersed in PBS at 4 °C for the following experiments.

### Characterization of the NPs

The morphologies of AuNP@mSiO_2_ and ZnO QDs were investigated through transmission electron microscopy (TEM, Tecnai G220, FEI, Holland) and scanning electron microscope (SEM, Sirion 200, FEI, Holland). ZEN 3600 instrument (Malvern, U.K.) was used to analyze zeta potential. Fourier transform infrared (FT-IR) spectra with the frequency ranging from 4000 to 400 cm^-1^ were recorded on a Bruker Tensor FT-IR spectrometer (VERTEX 70, Germany). UV absorbance spectra were observed with UV 2550 spectrophotometer (UV-1801, Beijing Rayleigh Analytical Instrument). The surface area and pore size distribution were determined by Brunauer-Emmett-Teller (BET) (TriStar II 3020, Alpha Technologies, USA) and Barrett-Joyner-Halenda (BJH) methods. The removal of CTAB in AuNP@SiO_2_ was identified by Thermogravimetric analyzer (TGA, Pyris1, Perkinelmer, USA).

### Cell lines and animals

Mouse melanoma cell line B16/F10 was purchased from American Type Culture Collection (ATCC) and cultured in Dulbecco's Modified Eagle medium (DMEM) supplemented with 10% fetal bovine serum (Gibco) and 100 U/mL penicillin/streptomycin (Hyclone, USA). Female C57bl/6 normal mice (5 weeks) were purchased from the Institute of Laboratory Animal Science, Beijing. During animal experiments, the Chinese law and guidelines of the local Ethical Committee on Animal Handling were followed, and all the mice were kept under specific pathogen-free conditions in the Animal Center of the Huazhong University of Science and Technology.

### DOX encapsulation and *in vitro* release

To prepare DOX-loaded AuNP@mSiO_2_-ZnO, DOX was mixed with AuNP@mSiO_2_ in phosphate buffer solution (PBS). After being stirred at a temperature of 37 °C overnight, AuNP@mSiO_2_@DOX was obtained by centrifugation and gently washed with PBS twice. The supernatant was combined to measure encapsulation efficiency of DOX by UV-vis measurement. In order to avoid DOX leakage of AuNP@mSiO_2_, ZnO-NH_2_ was covalently bonded on the surface of AuNP@mSiO_2_@DOX to cap the tunnels. The encapsulation efficiency (EE) is expressed according to the following formula: EE% = [(weight of loaded drug)/(weight of total drug in feed)] × 100 (Eq.1). To investigate the pH-triggered drug release, 10 mg of AuNP@mSiO_2_@DOX-ZnO was dispersed in 5 mL of buffer solutions (PBS buffer at pH 7.4 or acetate buffer at pH 5.0) and sealed in dialysis bags (molecular weight cutoff: 8000). The dialysis bags were submerged in 20 mL of corresponding buffer solutions and stirred at 37 °C for 48 h. The DOX released in the buffer was collected at selected time intervals and then analyzed by UV-vis spectroscopy.

### Cell uptake* in vitro* and *in vivo*

B16/F10 melanoma cells (1 × 10^5^ cells per well) were cultured in 6-well plates and treated with AuNP@mSiO_2_@DOX-ZnO for 2, 4 and 6 h, respectively. Subsequently, B16/F10 cells were washed and fixed with 4% paraformaldehyde for 20-30 min at room temperature. The fixed cells were stained with DAPI (dyes for nuclear staining) for 5 min and washed for three times with PBS. Then, these cells were subjected to confocal laser scanning microscope (CLSM, Olympus FV500, Japan) for detecting the endocytic ability of B16/F10 melanoma cells. Furthermore, to evaluate the cancer cell uptake of AuNP@mSiO_2_-ZnO or AuNP@mSiO_2_ *in vitro*, B16/F10 melanoma cells were incubated with AuNP@mSiO_2_-ZnO-Cy5 or AuNP@mSiO_2_-Cy5 (50 μg/mL) for 2, 4 and 6 h, respectively. Subsequently, the cells were washed with PBS and analyzed by flow cytometry (LSRII Orange, BD, USA). To evaluate the cancer cell uptake of AuNP@mSiO_2_-ZnO or AuNP@mSiO_2_ *in vivo*, AuNP@mSiO_2_-ZnO-Cy5 or AuNP@mSiO_2_-Cy5 were intratumorally injected to mice bearing subcutaneous melanoma. At 3 h post-injection, tumors were harvested and dissociated cell suspensions were obtained. The single-cell suspensions from tumors were resuspended with PBS. After incubated with anti-CD16/32, cells were stained with CD45 (30-F11), CD3e (145-2C11), F4/80(BM8), CD206(MR6F3), B220(RA3-6B2), CD11c (N418), PI staining solution and corresponding isotype controls (all from eBioscience). The staining protocols were as per the manufacturer's instructions (eBioscience). All the samples were subjected to flow cytometry (LSRII Orange, BD, USA) and data was analyzed with FlowJo software (Tree Star, Ashland, OR).

### Cytotoxicity and photothermal therapy *in vitro*

B16/F10 melanoma cells (1 × 10^4^ cells per well) were seeded in 96-well plates and cultured for 24 h. Then, the cells were incubated with varied concentration of AuNP@mSiO_2_, ZnO and AuNP@mSiO_2_-ZnO at different composition ratios for 24 h and the concentrations of AuNP@mSiO_2_ and ZnO were in accordance with AuNP@mSiO_2_-ZnO. After that, cell viability was evaluated using a CCK-8 kit (Dojindo, Japan). To further identify the photothermal effect of AuNP@mSiO_2_-ZnO, B16/F10 cells seeded onto 96-well plates for 24 h were washed three times with PBS and treated with PBS or AuNP@mSiO_2_-ZnO (50 μg/mL) for 6 h, and were then irradiated with a 655 nm laser (1.0 W/cm^2^) (MD-655-HS-1.8w-16060511, Changchun New Industries Optoelectronics Tech, China) for 0 min, 5 min and 10 min. After a 2 h incubation, the cells were washed and stained with calcein AM/PI (Invitrogen, Carlsbad, CA) and the visualization of cells (living or dead) was performed on a fluorescence microscope (IX71, Olympus, Japan).

### *In situ* tumor growth inhibition

Mice models of subcutaneous melanoma were intratumorally injected with AuNP@mSiO_2_-ZnO at dose of 60 μg per mouse and irradiated with a 655 nm laser with varied power doses and exposure times. The intra-tumoral temperature was monitored immediately after irradiation using an infrared fusion thermal imager (Ti95, Fluke, China). Furthermore, melanoma bearing mice, those in whom the tumors grew to ~ 5 mm in diameter (10 days after injecting the cells), were randomized into ten groups (n = 5): PBS, PBS + L ( a power density of 1.0 W/cm^2^ for 40 s), AuNP@mSiO_2_-ZnO, DOX, AuNP@mSiO_2_@DOX-ZnO, AuNP@mSiO_2_-ZnO + L (1.0 W/cm^2^, 40 s), AuNP@mSiO_2_@DOX-ZnO + L (1.0 W/cm^2^, 40 s), AuNP@mSiO_2_-ZnO + L (0.7 W/cm^2^, 40 s), AuNP@mSiO_2_-ZnO + L (1.0 W/cm^2^, 20 s) and AuNP@mSiO_2_-ZnO + L (1.3 W/cm^2^, 40 s). The dosages were kept constant, of which the dose of AuNP@mSiO_2_-ZnO or AuNP@mSiO_2_@DOX-ZnO were 60 μg per mouse and the dose of DOX was 6.72 μg. The amount of DOX in AuNP@mSiO_2_@DOX-ZnO solution was the same as that in the DOX group. All groups received intra-tumoral treatments every three days, for a total of three injections. The body weights and tumor volumes (width^2^ × length/2) were monitored every 2 days. All the tumors and major organs, including lung, heart, brain, liver, kidney and spleen were harvested on day 16. The tumors were weighed and photographed, one half of the tumors was fixed in 4% paraformaldehyde and the other half was processed into single-cell suspensions to analyze the tumor-infiltrating lymphocytes. In addition, all the tumors and lungs were sectioned and stained with Hematoxylin and Eosin (H&E) and Ki-67 to analyze histopathological abnormalities and evaluate the extent of metastases.

### Metachronous tumor growth and inhibition of lung metastasis

B16/F10 tumor cells were inoculated on the right flank of each mouse. One week later, either a second tumor was inoculated on the left flank or tumor cells were injected intravenously into the same mouse so as to mimic metastasis. In the following week, the primary tumor in all the groups (on the right flank) received intra-tumoral treatments every three days for a total of three injections. The body weights, and primary and second tumor volumes (width^2^ × length/2) were monitored every 2 days, and all the tumors were harvested on day 14^th^, when all the mice were sacrificed. The tumors were weighed and photographed, one half of the tumors was fixed in 4% paraformaldehyde and the other half was processed into single-cell suspensions and analyzed by flow cytometry. To investigate the inhibition of lung metastasis, all of the harvested lungs were weighed, photographed and metastatic nodules present in the lungs were counted. In addition, the lungs were also fixed in 4% paraformaldehyde and stained with H&E and S100 staining to evaluate the extent of metastasis.

### Flow cytometry analysis of anti-tumor immunity *in vivo*

Single-cell suspensions from tumors and tumor draining lymph nodes (TDLNs) were obtained via the methods mentioned above and resuspended with PBS. After incubating with anti-CD16/32, cells were stained with CD45 (30-F11), CD3e (145-2C11), CD4 (GK1.5), CD8 (53-6.7), CD44 (IM7), CD62L (MEL-14), PI staining solution and corresponding isotype controls (all from eBioscience). The staining protocols were as per the manufacturer's instructions (eBioscience). All the samples were detected by flow cytometry and data was analyzed with FlowJo software (Tree Star, Ashland, OR).

### Calreticulin exposure assay *in vitro*

B16/F10 melanoma cells were incubated with PBS, AuNP@mSiO_2_-ZnO (50 μg/mL) or AuNP@mSiO_2_@DOX-ZnO (the concentration was equal to AuNP@mSiO_2_-ZnO) for 6 h and irradiated with a 655 nm laser at 1.0 W/cm^2^ for 5 min. After further incubation for 4 h, the cells were collected, washed and incubated with Alexa Fluor 488-CRT antibody (ab196159, abcam) for 2 h, followed by staining with PI. Then, the samples were further analyzed with flow cytometry to explore calreticulin (CRT) expression on cell membranes.

### AuNP@mSiO_2_@DOX-ZnO induced maturation of DCs* in vivo*

A mouse model of subcutaneous melanoma was generated by inoculating 5×10^5^ B16/F10 cells/100 μL PBS into the dorsal flank of C57bl/6 normal mice. The tumors were then allowed to grow to ~ 5 mm in diameter. 3 days after treatment, TDLNs were harvested and DC maturation was analyzed by flow cytometric analysis. The cells were preincubated with anti-CD16/32 (clone 93, eBioscience, USA) to reduce non-specific binding to FcRs, which was followed by staining with CD11c (N418), CD80 (16-10A1) and CD86 (GL1) antibody (all from eBioscience, USA). Finally, the cells were stained with PI staining solution to exclude dead cells. Data was analyzed with FlowJo software (Tree Star, Ashland, OR).

### Western blotting

Briefly, B16/F10 tumor cells were treated with AuNP@mSiO_2_, AuNP@mSiO_2_-ZnO or AuNP@mSiO_2_@DOX-ZnO 50 μg/mL for 6 h and irradiated with 655 nm laser (1.0 W/cm^2^) for 5 min, after which the tumor cells were further cultured for 24 h or 48 h and the cell lysates were extracted. The following primary antibodies were used: Anti-HMGB1 (ab79823, Abcam), Anti-LC3B (ab51520, Abcam), Anti-p62 (ab56416, Abcam), anti-Baclin-1 (ab62557, Abcam), anti-GAPDH (ab181602, Abcam) and β-actin (C4, Santa Cruz).

### ELISA of HMGB1

B16/F10 tumor cells were incubated with PBS, AuNP@mSiO_2_, AuNP@mSiO_2_-ZnO or AuNP@mSiO_2_@DOX-ZnO 50 μg/mL for 6 h and irradiated with a 655 nm laser (1.0 W/cm^2^) for 5 min, after which the tumor cells were further cultured for 72 h to collect supernatant. The expression of HMGB1 was assayed using the ELISA method (kits purchased from Cusabio (CSB-E08225m).

### ATP release assay

B16/F10 melanoma cells were treated as indicated for 48 h. Subsequently, the supernatants were collected and the concentration of ATP was detected by a chemiluminescence ATP Determination Kit (A22066, Invitrogen, UK) according to the manufacturer's instructions.

### Transmission electron microscopy characterization of B16/F10 melanoma cells

The cells were treated as indicated. Then, the samples were collected and fixed with 2.5% phosphate-buffered glutaraldehyde. After washing with PBS, the cells were post-fixed with 1% OsO_4_, dehydrated and embedded in Spurr's resin. Thin sections (~50-70 nm) were cut on a UC6 ultramicrotome (Leica Microsystems, Wetzlar, Germany) and stained with 4% uranyl acetate and lead citrate. Finally, digital images were acquired with a Tecnai G2 12 transmission electron microscope (FEI, Hillsboro, OR, USA).

### Statistical analysis

The sample sizes were chosen based on preliminary data from our pilot experiments and previous publications in the literature 3,8,10. All quantitative data was presented as mean ± standard deviation. Statistical analysis was performed with Prism 5.0 software (GraphPad Software). An unpaired Student's *t*-test and a two-way ANOVA with Bonferroni multiple comparisons post-test were performed to compare the data between two groups. Statistical significance is indicated as *P < 0.05, **P < 0.01, ***P < 0.001.

## Results and Discussion

### Synthesis and characterization of AuNP@mSiO_2_ based nanocomposites

The schematic procedure for preparing AuNP@mSiO_2_@DOX-ZnO is shown in Figure 1A. Initially, uniform gold NPs, serving as a core, were prepared with a diameter of ∼18 nm. AuNP@mSiO_2_ core-shell NPs with a uniform thickness of ~27 nm were then obtained, in which CTAB functioned as a stabilizer and as a soft template for the formation of mesoporous silica in a sol-gel reaction 24. Uniform spherical AuNP@mSiO_2_ with a size of ∼72 nm and a dendritic mesoporous structure were clearly observed on transmission electron microscopy (TEM) and scanning electron microscopy (SEM) images (Figure 1C-D). After removal of CTAB micelles from the mesoporous shell by ion exchange method, AuNP@mSiO_2_ was grafted with 2, 5-Furandione, dihydro-3-[3-(tri ethoxy silyl) propyl] (TESPA) to provide carboxylic groups. Subsequently, aminopropyltriethoxysilane (APTES)-modified ZnO QDs were fabricated according to a previously reported method 25. The obtained ZnO QDs were well-dispersed with an ultra-small size (∼5 nm) (Figure 1B). Then, TESPA-grafted AuNP@mSiO_2_ (AuNP@mSiO_2_-COOH) were coupled with APTES-modified ZnO QDs through amide bonds. TEM images indicated that ZnO QDs successfully capped the channels of AuNP@mSiO_2_-COOH (Figure 1E). Meanwhile, Brunauer-Emmett-Teller (BET) and Barrett-Joyner-Halenda (BJH) analyses showed that the pore size, BET surface area and total pore volume of AuNP@mSiO_2_-ZnO were lower than those of AuNP@mSiO_2_ (Figure S1E and Table S1)_._ This indicated that ZnO QDs were large enough to cap the channels of AuNP@mSiO_2_ (pore size: ~2.8 nm), preventing the leakage of loaded drugs. Additionally, an energy dispersive X-ray spectroscopy (STEM-EDX) mapping for Zn, Si, O and Au was also used to confirm the presence of all the constituent elements in AuNP@mSiO_2_-ZnO nanocomposites (Figure S1A-D). UV-vis absorption spectra, wide-angle X-ray diffraction (XRD) patterns and zeta potentials of AuNP@mSiO_2_-ZnO further confirmed that AuNP@mSiO_2_-ZnO nanocomposites had been successfully synthesized (Figure 1F, Figure S1F and Figure S2).

### Photothermal properties, drug loading and pH-triggered release

To demonstrate the photothermal effect, temperature variation of the aqueous solution containing AuNP@mSiO_2_-ZnO nanocomposites at various concentrations was detected by irradiating with a 655 nm laser, using varying power densities *in vitro*. Upon laser irradiation (2.0 W/cm^2^) for 5 min, AuNP@mSiO_2_-ZnO with a concentration of 0.75 mg/mL showed a significant temperature increase from 25 °C to 45 °C (Figure 1G-H). These results suggest that AuNP@mSiO_2_-ZnO nanocomposites have high photothermal conversion ability. In addition, we evaluated the potential of AuNP@mSiO_2_-ZnO nanocomposites in *in vitro* drug encapsulation. Firstly, DOX was incubated with AuNP@mSiO_2_-COOH for 24 h. After DOX encapsulation, APTES-modified ZnO QDs were added to the above solution to cap the tunnels of AuNP@mSiO_2_-COOH through formation of amide bonds. The encapsulation efficiency measured as high as 33.89 ± 1.64 % when the ratio of AuNP@mSiO_2_ and ZnO was kept at 5:1 (Figure 1I), which can be ascribed to the blockage of pores with ZnO QDs, thus effectively preventing the leakage of the entrapped DOX.

To further explore the pH-responsive behavior of ZnO QDs, AuNP@mSiO_2_-ZnO nanocomposites were incubated in an acetate buffer (pH 5.0) followed by phosphate-buffered saline (PBS, pH 7.4) for 12 h, respectively. The fluorescence signal of ZnO QDs was monitored by fluorescence spectroscope (λ_ex_ = 350 nm). Generally, ZnO QDs are stable at a pH value of 7.4 with the maximum fluorescence emission observed at 530 nm, and quickly decompose to ionic Zn^2+^ at pH values < 5.5 19. As expected, AuNP@mSiO_2_-ZnO nanocomposites were luminescent at pH 7.4, while no luminescence signals were observed in acetate buffer (pH 5.0), indicating that the ZnO QDs could be easily uncapped in the acidic microenvironment (Figure 1J). To further verify this pH-triggered, drug release behavior observed with AuNP@mSiO_2_@DOX-ZnO, the cumulative release kinetics of the nanocomposites in solutions of pH 7.4 and pH 5.0 at 37 °C were evaluated* in vitro*. The results indicated that 60.26 ± 4.31% of DOX was released in an acetate buffer (pH 5.0) within 48 h and reached a plateau, while only 8.51 ± 1.35% of DOX was released in the PBS control (pH 7.4) (Figure 1K). Therefore, it is expected that the ZnO QDs can be readily uncapped in the acidic intracellular compartments of cancer cells to further trigger DOX release from AuNP@mSiO_2_-ZnO.

### Uptake, cell viability and photothermal performance

**Figure 2:**
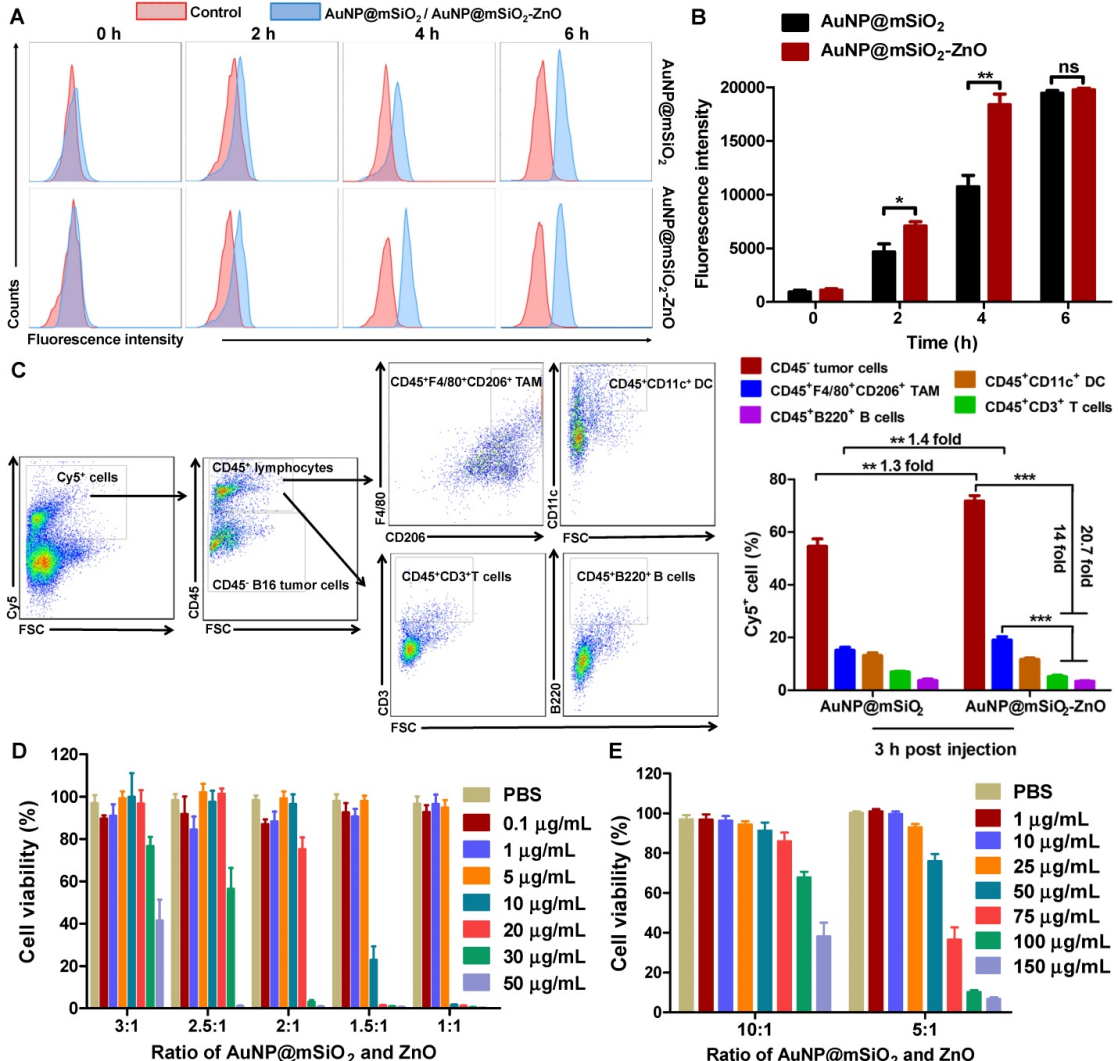
Uptake and *in vitro* cell viability of AuNP@mSiO_2_-ZnO. (A, B) Cellular uptake of AuNP@mSiO_2_-Cy5 or AuNP@mSiO_2_-ZnO-Cy5 by B16/F10 melanoma cells* in vitro*. The cells were incubated for 2, 4 and 6 h, respectively. AuNP@mSiO_2_ and AuNP@mSiO_2_-ZnO accumulations, represented by the fluorescence intensity of Cy5, were determined by (A) flow cytometry, and (B) the mean fluorescence intensity. (C) Cellular uptake and internalization of AuNP@mSiO_2_-ZnO nanocomposite *in vivo*. The proportions of B16/F10 melanoma cells, TAM, DC, T and B cells that internalized AuNP@mSiO_2_-ZnO-Cy5 or AuNP@mSiO_2_-Cy5 were analyzed for 3 h after intratumor injection. (D, E) Cell viability of B16/F10 melanoma cells treated with various concentrations of AuNP@mSiO_2_-ZnO at different composition ratios of AuNP@mSiO_2_ to ZnO for 24 h. As the ratio of AuNP@mSiO_2_ to ZnO was increased, the cytotoxicity of AuNP@mSiO_2_-ZnO to B16/F10 melanoma cells decreased gradually in a ZnO QDs dose-dependent manner. AuNP@mSiO_2_-ZnO began to exert cytotoxicity when the ratio for AuNP@mSiO_2_ and ZnO was 5:1 at 50 µg/mL. Results are representative of at least three independent experiments. Data were given as mean ± SD. The error bars represent the standard deviation. Statistical significance was calculated by the t-test. **P < 0.01, ***P < 0.001. TAM represents tumor-associated macrophages; DC refers to dendritic cell.

To investigate the uptake of AuNP@mSiO_2_@DOX-ZnO nanocomposites, B16/F10 melanoma cells were incubated with AuNP@mSiO_2_@DOX-ZnO for varying times and then analyzed by a confocal laser scanning microscope (CLSM). CLSM images showed that after 4 h of incubation, the intense red fluorescent signal of DOX became visible around cellular nuclei, reaching its peak intensity at 6 h (Figure S3). This suggests that the AuNP@mSiO_2_-ZnO nanocarrier can efficiently and rapidly deliver antitumor drugs into cancer cells. We further compared the endocytic ability of B16/F10 melanoma cells for AuNP@mSiO_2_-ZnO-Cy5 and AuNP@mSiO_2_-Cy5 using flow cytometry. In the case of AuNP@mSiO_2_-ZnO, the intensity of fluorescence was stronger than that of AuNP@mSiO_2_ at 2 h (1.5-fold) and 4 h (1.7-fold), although they were almost equal at 6 h (Figure 2A-B), suggesting that AuNP@mSiO_2_-ZnO was preferentially endocytosed by melanoma cells. Next, we explored the *in vivo* uptake of AuNP@mSiO_2_ or AuNP@mSiO_2_-ZnO nanocomposite in mice bearing subcutaneous melanomas. At 3 h post-injection, the AuNP@mSiO_2_-ZnO nanocomposites were predominantly taken up by B16/F10 melanoma cells (71.85%) when compared with uptake by CD45^+^CD3^+^ T cells (5.13%) and CD45^+^B220^+^ B cells (3.46%), respectively (Figure 2C). Moreover, compared with AuNP@mSiO_2_, AuNP@mSiO_2_-ZnO was more efficiently phagocytosed by B16/F10 tumor cells (Figure 2C and Figure S4). The preferential phagocytic ability of cancer cells towards ZnO might be ascribed to the positive charge derived from ZnO and their stronger phagocytic function 19,26-30. Unexpectedly, although the majority of the AuNP@mSiO_2_-ZnO nanocomposites were engulfed by B16/F10 tumor cells, 19.13% of them were also taken up by CD45^+^F4/80^+^CD206^+^ tumor-associated macrophages (TAMs) (Figure 2C). Furthermore, B16/F10 melanoma cells, TAMs, dendritic cells (DCs), T cells and B cells were also gated and assessed for their endocytic ability for AuNP@mSiO_2_-ZnO-Cy5. In a tumor microenvironment, the majority of B16/F10 melanoma cells (70.78 ± 2.07%) and TAMs (84.73 ± 2.16%) phagocytosed AuNP@mSiO_2_-ZnO-Cy5 (Figure S5A). TAMs are one of the most abundant tumor-infiltrating leukocytes and exhibit tumor-promoting properties 31. TAMs act as key players in angiogenesis, tumor cell invasion and metastasis progression by secreting hypoxia-inducible factors (HIFs), vascular endothelial growth factor (VEGF) and matrix metalloproteinases (MMPs) 31. TAMs also sabotage anti-tumor immunity by impairing T cell activation and promoting immune suppression by producing IL-10, CC-chemokine ligand 22 (CCL22) and TGF-β 31. The preferential uptake of AuNP@mSiO_2_-ZnO nanocomposites by TAMs may be derived from them exhibiting an M2 phenotype, which is linked to a strong phagocytic function 32. Combined with a pH-triggered DOX release, the preferential uptake of AuNP@mSiO_2_@DOX-ZnO nanocomposites by B16/F10 melanoma cells and TAMs may further enhance their anti-tumor efficacy.

Previous studies showed that Zn^2+^ release from the dissolution of ZnO QDs had potential to generate reactive oxygen species (ROS) and enhance the cytotoxic effect 29,33,34. Therefore, the optimal ratio of ZnO QDs and AuNP@mSiO_2_ needs to be ascertained so as to balance the drug-loading efficiency with systemic toxicity of AuNP@mSiO_2_-ZnO nanocomposites. To confirm the cytotoxic effect of the different components in AuNP@mSiO_2_-ZnO nanocomposites, B16/F10 melanoma cells were incubated separately with AuNP@mSiO_2_, and with ZnO and AuNP@mSiO_2_-ZnO at different composition ratios for 24 h. At varying concentrations between 0.01 to 500 μg/mL (Figure S5B), there was no cytotoxicity observed in the AuNP@mSiO_2_ group. However, for the ZnO QDs, cell viability was notably decreased to 68.91 ± 6.94% at a concentration of 10 μg/mL (Figure S5C). Similarly, when the ratio of AuNP@mSiO_2_ to ZnO was increased, the cytotoxicity of AuNP@mSiO_2_-ZnO to B16/F10 melanoma cells decreased gradually in a ZnO QDs dose-dependent manner (Figure 2D-E). With a 5:1 ratio of AuNP@mSiO_2_ to ZnO, AuNP@mSiO_2_-ZnO began to exert cytotoxicity and decreased cell viability to 75.97 ± 3.54% at a concentration of 50 μg/mL (Figure 2E). Therefore, the AuNP@mSiO_2_-ZnO nanocomposite with a 5:1 ratio of AuNP to ZnO was selected as an efficient nanocarrier for the combined therapy of melanoma in the following *in vitro* experiment.

Furthermore, to demonstrate the photothermal effect of AuNP@mSiO_2_-ZnO* in vitro*, B16/F10 cells were incubated with AuNP@mSiO_2_-ZnO at a concentration of 50 μg/mL for 4 h (the concentration of AuNP@mSiO_2_ and ZnO was 50 μg/mL and 10 μg/mL, respectively). Clearly, compared with the PBS + L (Laser irradiation) control group, the nanocomposites showed significant cytotoxicity when irradiated with a 655 nm laser at a power density of 1.0 W/cm^2^ for 10 min (Figure S6), indicating that AuNP@mSiO_2_-ZnO could further enhance the cytotoxic effect with its added photothermal effect.

### AuNP@mSiO_2_@DOX-ZnO effectively inhibits the progression of melanoma* in situ* by eliciting antitumor immunity

**Figure 3:**
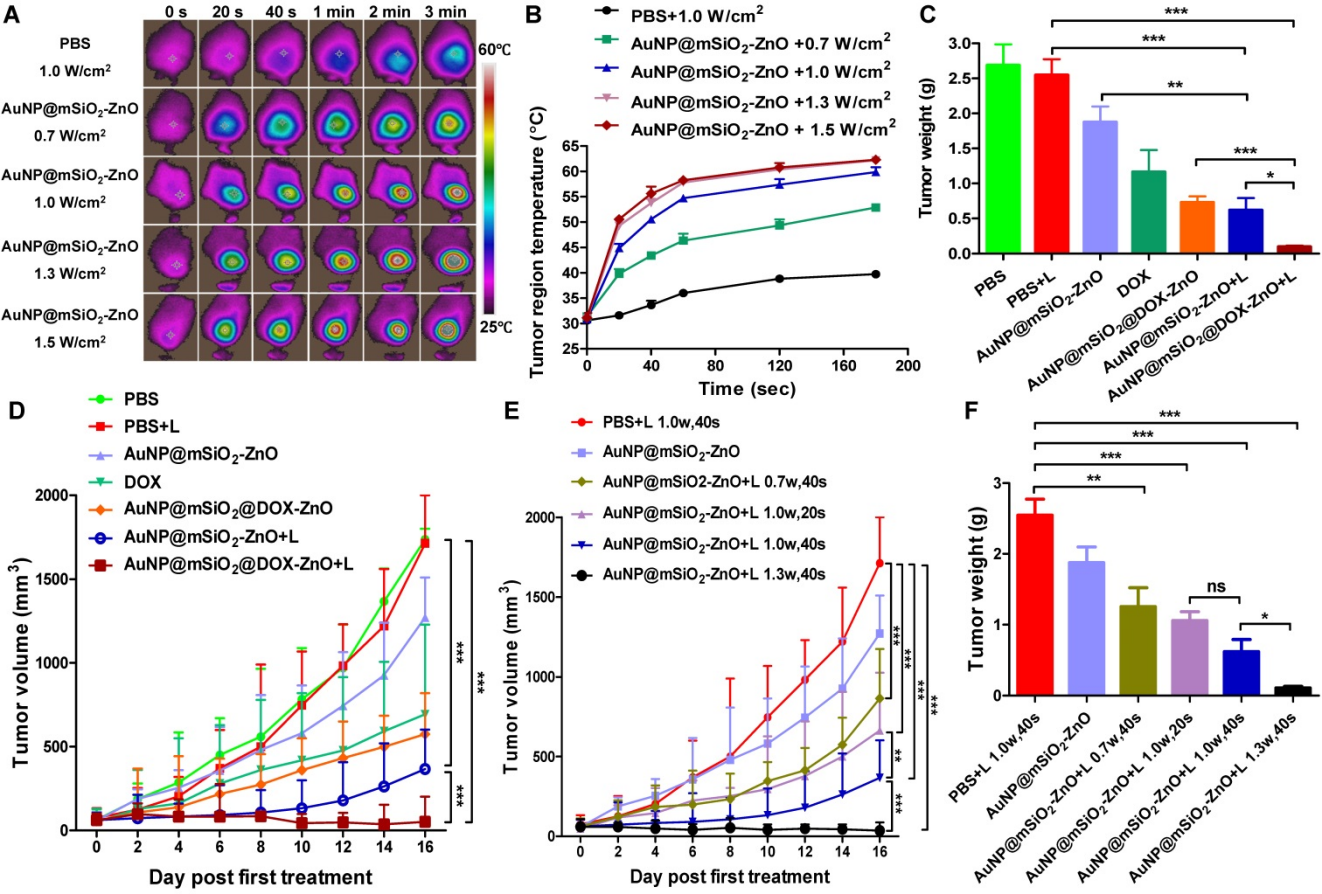
*In vivo* anti-tumor effect on murine melanoma after intratumoral injection. (A, B) Representative thermographic images and regional temperature changes of tumor-bearing mice treated with PBS or AuNP@mSiO_2_-ZnO and irradiated by a 655 nm laser with varying time and power densities. (C, D) The tumor weight and tumor volume of mice after different treatments as indicated. The tumors in laser irradiation groups were irradiated with laser (1.0 W/cm^2^) for 40 s. (E, F) Tumor-bearing mice were injected intratumorally with PBS or AuNP@mSiO_2_-ZnO and irradiated with various time and power intensities. The tumor volumes of each group and tumor weight at the end of experiment were measured. L refers to laser irradiation. Statistical significance was calculated by the t-test. *P < 0.05, **P < 0.01, ***P < 0.001. Data was presented as mean ± SD (n = 5 mice per group). The error bars represent the standard deviation.

To evaluate the *in vivo* anti-tumor efficiency, AuNP@mSiO_2_-ZnO nanocomposites were intra-tumorally injected into mice bearing subcutaneous melanoma. At 3 h post-injection, the fluorescent signal of AuNP@mSiO_2_-ZnO-Cy5 reached its maximum level in the tumor (Figure S7). Then, the entire tumor region was irradiated by a 655 nm laser using varied time intervals and power densities. The average temperature of the tumor region quickly rose to 50.57 ± 0.74 °C, 49.27 ± 0.60 °C and 50.53 ± 0.71 °C in groups with irradiation of 1.0 W/cm^2^ for 40 s, 1.3 W/cm^2^ for 20 s and 1.5 W/cm^2^ for 20 s, respectively (Figure 3A-3B), which are high enough to efficiently ablate tumors 35. With further increases in the laser irradiation time, the temperature increased to 60 °C and reached a near plateau 3 min later (Figure 3A-B).

Next, mice bearing subcutaneous melanoma were randomly divided into 7 groups (n=5) based on the contents of the intra-tumoral injections (PBS, AuNP@mSiO_2_-ZnO with and without DOX, and free DOX at the same doses) with or without laser irradiation. In the case of laser irradiation groups, at 3 h post-injection, the tumor regions were irradiated at 1.0 W/cm^2^ for 40 s. After treatment (once every three days) for a total of three cycles, all tumors in the group of AuNP@mSiO_2_@DOX-ZnO +L were almost completely eliminated at day 10. At day 16, compared to the PBS + L control group, AuNP@mSiO_2_@DOX-ZnO exhibited a 95.5% reduction in tumor volume and a 96.2% reduction in tumor weight. Meanwhile, significant suppression of tumor growth was also observed in the case of AuNP@mSiO_2_-ZnO-based PTT (78.3% and 75.7% decrease in tumor size and weight) and DOX (55.4% and 56.8% decrease in tumor size and weight), but this was not as marked as that in the AuNP@mSiO_2_@DOX-ZnO group (Figure 3C-D and Figure S8). Thus, we suggest that combined therapy exhibits greater efficiency in inhibition of tumor growth, compared with AuNP@mSiO_2_@DOX-ZnO and AuNP@mSiO_2_-ZnO-based PTT monotherapies.

To further investigate the PTT therapy potential of AuNP@mSiO_2_-ZnO and explore the optimal parameters of inhibition of tumor growth *in vivo*, we evaluated the anti-tumor effects of AuNP@mSiO_2_-ZnO irradiated with various exposure times and power intensities. We established the group under treatment with 1.0 W/cm^2^ for 40 s *in vivo*, as the comparison group and then used power intensities between 0.7 or 1.3 W/cm^2^ while keeping the exposure time constant. We repeated the set of experiments with an exposure time of 20s and a power density of 1.0 W/cm^2^. Compared to the PBS + L control group (1.0 W/cm^2^ for 40 s), both the tumor volumes and tumor weights declined significantly in all the irradiated groups (Figure 3E-F). Furthermore, compared with the group treated at 1.0 W/cm^2^ for 40 s (78.3% and 75.7% decrease in tumor size and weight), the groups treated with 0.7 W/cm^2^ for 40 s (50.2% and 50.6% decrease) and 1.0 W/cm^2^ for 20 s (58.3% and 58.4% decrease) exhibited a lesser reduction in tumor volume and tumor weight. We observed the most significant decrease in tumor volumes (97.5 %) and weights (95.7 %) at a power density of 1.3 W/cm^2^ for 40 s, thus exhibiting a time- and power intensity- dependent profile. Consistently, hematoxylin and eosin (H&E) staining and immunohistochemistry (IHC) staining of Ki-67 of tumor slices were performed to visualize the anti-tumor effects. On H&E staining, cellular damage within tumors was observed with AuNP@mSiO_2_-ZnO in all the irradiation groups. The group treated with 1.3 W/cm^2^ for 40 s exhibited the most severe degree of cellular damage, while the PBS + L group retained normal cellular morphology (Figure S9). IHC staining showed no noticeable metastatic sites in the lung tissue in all the groups of AuNP@mSiO_2_-ZnO that were irradiated (Figure S9). This data indicates that AuNP@mSiO_2_-ZnO-based PTT has the potential to inhibit melanoma growth, in a time- and power intensity-dependent pattern.

**Figure 4:**
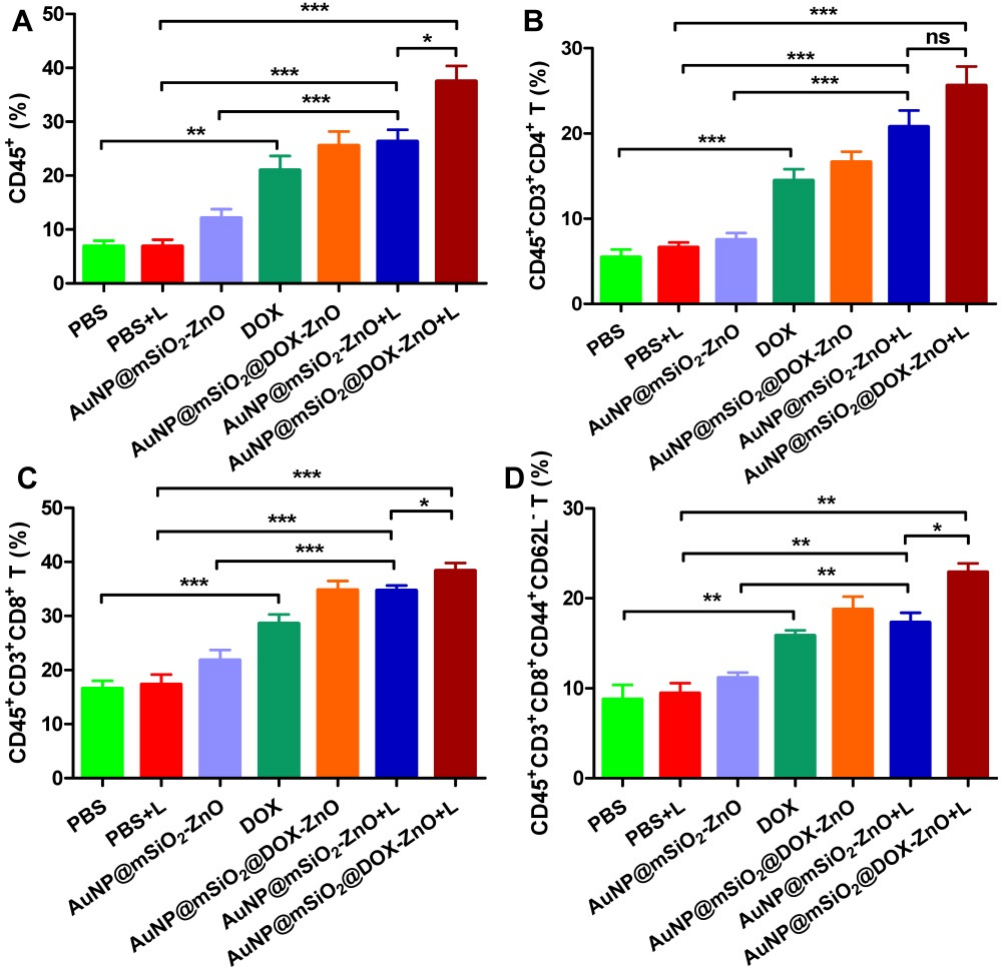
AuNP@mSiO_2_@DOX-ZnO with laser irradiation induced antitumor immune responses. The percentages of CD45^+^ leukocytes (CD45^+^PI^-^) (A), CD4^+^ T cells (CD45^+^CD3^+^CD4^+^PI^-^) (B), CD8^+^ T cells (CD45^+^CD3^+^CD8^+^PI^-^) (C) and activated CD8^+^ T cells (CD45^+^CD3^+^CD8^+^ CD44^+^CD62L^-^PI^-^) in tumor (D) were analyzed by flow cytometric analysis. Statistical significance was calculated by the t-test. *P < 0.05, **P < 0.01, ***P < 0.001. Data was presented as mean ± SD (n=5). The error bars represent the standard deviation. L refers to laser irradiation.

Numerous studies show that PTT with multiple NPs (*e.g.*, CuS, graphene oxide, ICG or carbon nanotubes) can ablate tumor cells via inducing hyperthermia, and can elicit an antitumor immune response 4, 36. Taking these distinct, *in vivo* antitumor effects into consideration, we questioned whether the inhibition of tumor growth had partially resulted from an antitumor immune response induced by AuNP@mSiO_2_@DOX-ZnO and AuNP@mSiO_2_-ZnO-based PTT. We found that DOX, AuNP@mSiO_2_-ZnO + L, and AuNP@mSiO_2_@DOX-ZnO + L groups exhibited significantly increased proportions of tumor infiltrating CD45^+^ T cells (20.97 ± 2.66%, 26.33 ± 2.18% and 37.46 ± 2.90%), compared to the PBS + L control group (6.87 ± 1.06%). Specifically, the percentages of CD4^+^ T cells (14.47 ± 1.34%, 20.75 ± 1.95% and 25.60 ± 2.25%), CD8^+^ T cells (28.54 ± 1.72%, 34.70 ± 0.96% and 38.35 ± 1.46%) and activated CD8^+^CD44^+^CD62L^-^ T cells (15.85 ± 0.59%, 17.30 ± 1.09% and 22.90 ± 0.98%) compared to the PBS+ L control group (CD4^+^ T cells, 6.62 ± 0.59%; CD8^+^ T cells, 17.33 ± 1.84%; activated CD8^+^CD44^+^CD62L^-^ T cells, 9.44 ± 1.16%) increased significantly across these three groups (Figure 4, Figure S10 and Figure S11). Furthermore, the AuNP@mSiO_2_@DOX-ZnO + L treatment induced a stronger anti-tumor immune response, compared to the DOX and AuNP@mSiO_2_-ZnO + L groups (Figure 4, Figure S10 and Figure S11). In summary, the AuNP@mSiO_2_-ZnO-based PTT alone can initiate a significant anti-tumor immune response and addition of DOX exhibits a further synergistic effect. Previous reports have noted that when irradiating with a 655 nm laser, compared with gold nanomaterial-mediated PTT (including gold nanospheres, nanorods, nanoshells or nanocluster based combined therapies) 37, AuNP@mSiO_2_-ZnO nanocomposites are responsive to relatively lower exposure times and power intensities. This efficacy might be partially related to the ability to evoke an enhanced antitumor immune response.

### ZnO triggered anti-tumor immunity by inducing immunogenic cell death

**Figure 5:**
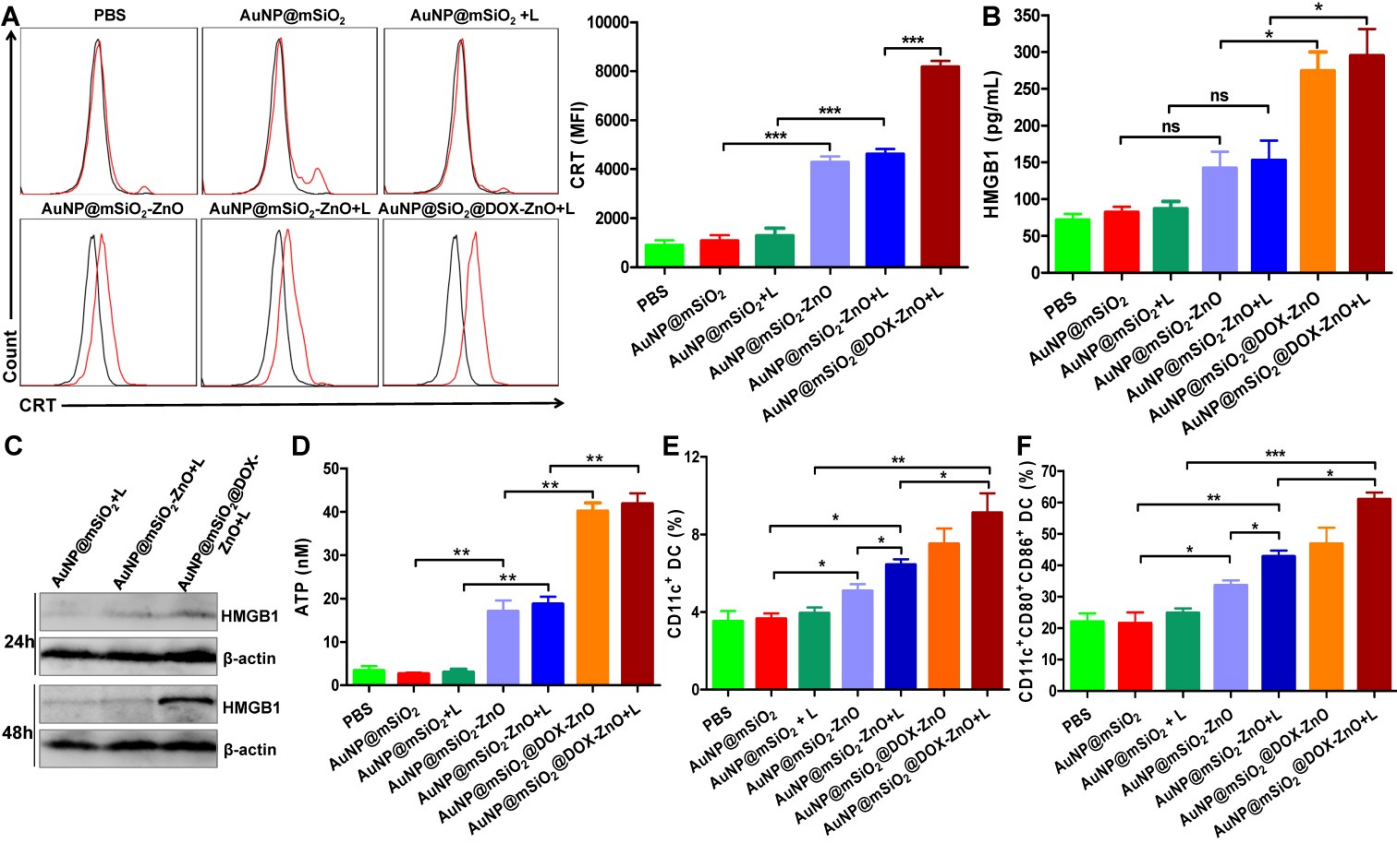
AuNP@mSiO_2_@DOX-ZnO induced ICD by promoting tumor cells to release DAMP and DC maturation *in vitro* and *in vivo*. (A) Flow cytometric analysis of the CRT exposure on the surface of B16/F10 melanoma cells after treatment with PBS, AuNP@mSiO_2_, AuNP@mSiO_2_-ZnO or AuNP@mSiO_2_@DOX-ZnO with or without irradiation by 655 nm laser at 1.0 W/cm^2^ for 5 min as indicated. The red line indicates Alexa Fluor 488-CRT, and the black line indicates isotype. (B, D) HMGB1 and ATP secretion in culture supernatants after treatment for 72 h and 48 h, respectively. (C) The expression of intracellular HMGB1 was detected after B16/F10 melanoma cells were treated as indicated for 24 h and 48 h. (E, F) The percentages of CD11c^+^DCs and CD11c^+^CD80^+^CD86^+^DCs in TDLNs of tumor-bearing mice after treatment for 72 h were identified by flow cytometric analysis (n = 3). L refers to laser irradiation. Results are representative of at least three independent experiments. Statistical significance was calculated by the *t*-test. Data was presented as mean ± SD. The error bars represent the standard deviation. *P < 0.05, **P < 0.01. ns refers to not significant. TDLNs: tumor draining lymph nodes.

We found that AuNP@mSiO_2_@DOX-ZnO + L exhibited excellent anti-tumor effect *in vivo* with an enhanced immune response. Investigation into the mechanisms behind the trigger for this anti-tumor immunity remains an interesting pursuit. Notably, recent studies have found that certain chemotherapies 38, PTT 39, photodynamic therapy 40-42 and other treatments 43-45 can initiate an antitumor immune response by inducing tumor cells to undergo immunogenic cell death (ICD). Furthermore, cancer cells undergoing ICD can release immunostimulatory damage-associated molecular patterns (DAMPs) that act as potent danger signals, including calreticulin (CRT) exposure on the surface of tumor cells, release of large amounts of high-mobility group box 1 (HMGB1) or extracellular secretion of adenosine triphosphate (ATP). These danger signals activate various receptors that are expressed on the surface of dendritic cells (DCs) and stimulate the presentation of tumor antigens to T cells, further activating the immune system 46,47. Therefore, we set out to ascertain how and if the different components of AuNP@mSiO_2_@DOX-ZnO could induce ICD by promoting tumor cells to release DAMPs. We found that compared to PBS and AuNP@mSiO_2_, AuNP@mSiO_2_-ZnO could induce CRT exposure to a significantly higher degree (Figure 5A). Compared with AuNP@mSiO_2_-ZnO, the CRT mean fluorescence intensity (MFI) did not change significantly in the AuNP@mSiO_2_-ZnO + L group. However, the MFI was enhanced when treated with AuNP@mSiO_2_@DOX-ZnO + L, indicating that both ZnO NPs and DOX but not the AuNP@mSiO_2_-based PTT could induce CRT exposure on the surface of B16/F10 melanoma cells (Figure 5A). Furthermore, as shown in Figure 5B-D, ZnO NPs can increase the release of ATP, and DOX has the ability of inducing secretion of HMGB1 and ATP release while AuNP@mSiO_2_-based PTT does not exhibit these properties. In summary, the above data indicates that ZnO NPs can induce CRT expression and ATP release. Also, DOX results in the expressions of CRT, HMGB1 and ATP, which has been well established in previous reports 48,49. Alternatively, AuNP@mSiO_2_-based PTT predominantly destroys tumors directly but not by releasing DAMPs.

Considering that ICD can further stimulate the activation of DCs and induce tumor-specific T-cell immune responses, we then investigated whether AuNP@mSiO_2_@DOX-ZnO under laser irradiation could also stimulate the maturation and activation of DCs within TDLNs* in vivo*. 3 days after treatment, TDLNs were collected and the percentage and maturation of DC was analyzed by flow cytometry. The percentages of CD11c^+^ DC and CD11c^+^CD80^+^CD86^+^ DC were significantly increased in the AuNP@mSiO_2_@DOX-ZnO + L group (9.12 ± 1.01%, 57.63 ± 5.13%) , compared to the AuNP@mSiO_2_ + L control group (3.94 ± 0.29%, 24.73 ± 1.55%) (Figure 5E-F), indicating that combined therapy of AuNP@mSiO_2_@DOX-ZnO + L could induce the migration of DCs into TDLNs and promote the maturation of DCs. Specifically, the percentages of CD11c^+^ DC and CD11c^+^CD80^+^CD86^+^ DC in AuNP@mSiO_2_-ZnO (5.08 ± 0.36%, 33.57 ± 1.64%) were higher than that in the AuNP@mSiO_2_ group (3.65 ± 0.27%, 21.47 ± 3.55%), while they were lower than that in the AuNP@mSiO_2_-ZnO + L group (6.43 ± 0.29%, 42.8 ± 1.91%) (Figure 5E-F). Thus, we speculate that ZnO acts as an immunoadjuvant by inducing ICD, which stimulating the activation of DCs, and overall triggering an effective tumor-specific immune response.

ICD is a specific form of cell death, which involves in multiple forms of cell death, including apoptosis, autophagy, necroptosis and pyroptosis 50. We found that compared with the PBS group, AuNP@mSiO_2_ + L and DOX could induce greater cell apoptosis and necrosis (Figure S12). Nevertheless, compared to AuNP@mSiO_2_, AuNP@mSiO_2_-ZnO only slightly decreased cell viability (76 ± 3.2%) (Figure S12). To further study how ZnO NPs trigger ICD, we identified the forms of cell death induced by AuNP@mSiO_2_-ZnO using a TEM. Unexpectedly, typical morphological features of autophagy (autophagosomes and autolysosomes) were observed in the AuNP@mSiO_2_-ZnO + L group (Figure S13), rather than in the AuNP@mSiO_2_ + L group. This result indicates that autophagic death might contribute to the ICD-inducing effect of ZnO NPs. To further confirm that autophagic death was responsible for ZnO-induced cytotoxicity, we examined the expressions of autophagy-related proteins LC3B, p62 and autophagy-related gene Beclin 1 by western blotting. When autophagy occurs, LC3B is transformed from a cytosolic LC3B-I to a membrane form LC3B-II, while p62 is degraded 51. As shown in Figure S14B, compared to AuNP@mSiO_2_, AuNP@mSiO_2_-ZnO significantly increased the expressions of LC3B-II and Beclin 1, and decreased the expression of p62. Similar results were obtained by TEM investigation (Figure S14C), which indicates that ZnO could trigger autophagic death in melanoma. As mentioned above, ZnO NPs might cause cell death via the production of ROS 29,33,34. Similarly, we also demonstrated that ZnO could trigger autophagic death via ROS production in melanoma (Figure S14). Meanwhile, blockage of ROS resulted in significant reduction of CRT exposure and ATP release in B16/F10 melanoma cells (Figure S15). Previous reports suggest that CRT exposure during ICD is an earlier process and depends on properties of ER stress and ROS production induced by immunogenic apoptosis 52. However, the secretion of extracellular HMGB1 and ATP was found to be excreted from cells undergoing late stage of apoptosis or autophagy 52. Therefore, we speculate that ZnO NPs might induce ICD by triggering autophagic death and apoptosis by promoting ROS production.

### AuNP@mSiO_2_@DOX-ZnO-based combined therapy prevents metastatic tumors from growing by activating anti-tumor immune responses

**Figure 6:**
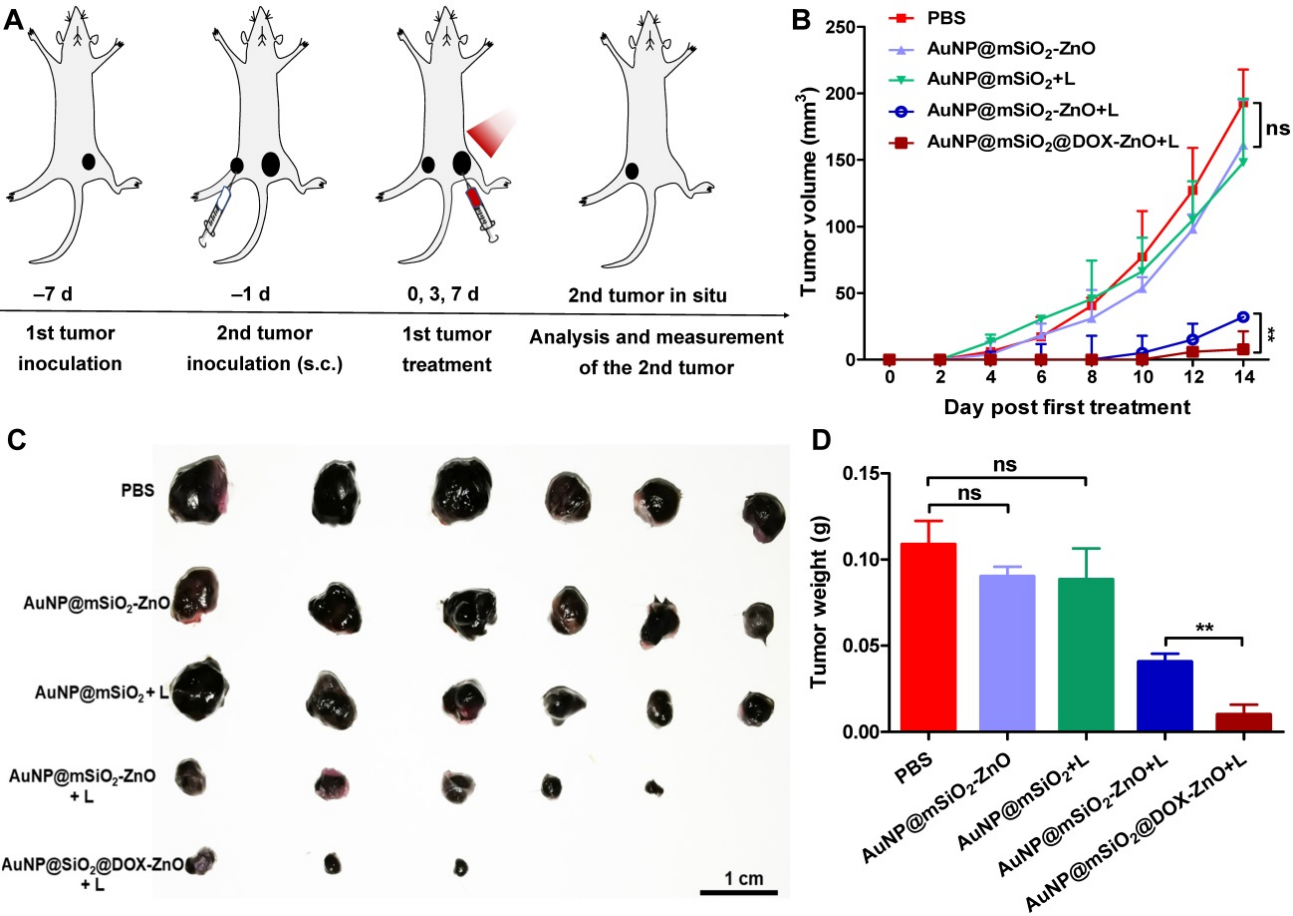
AuNP@mSiO_2_@DOX-ZnO prevents secondary tumors grow. (A) Schematic illustration exhibiting the design of animal experiments of metachronous tumors. (B) Bilateral subcutaneous melanoma mice models were established. The 1^st^ tumor in all the groups received intratumoral treatments every three days for a total of three injections and the tumor volumes of the secondary tumors in each group were measured for 14 days after the 1^st^ tumor treatment. (C, D) All the tumors were harvested on day 14. Photographs of the secondary tumors after excision from bilateral subcutaneous melanoma mice model were taken and tumor weight of the secondary tumors were measured (n=6). L refers to laser irradiation. Statistical significance was calculated by the t-test. Data was presented as mean ± SD. The error bars represent the standard deviation. ns refers to not significant, **P < 0.01.

**Figure 7:**
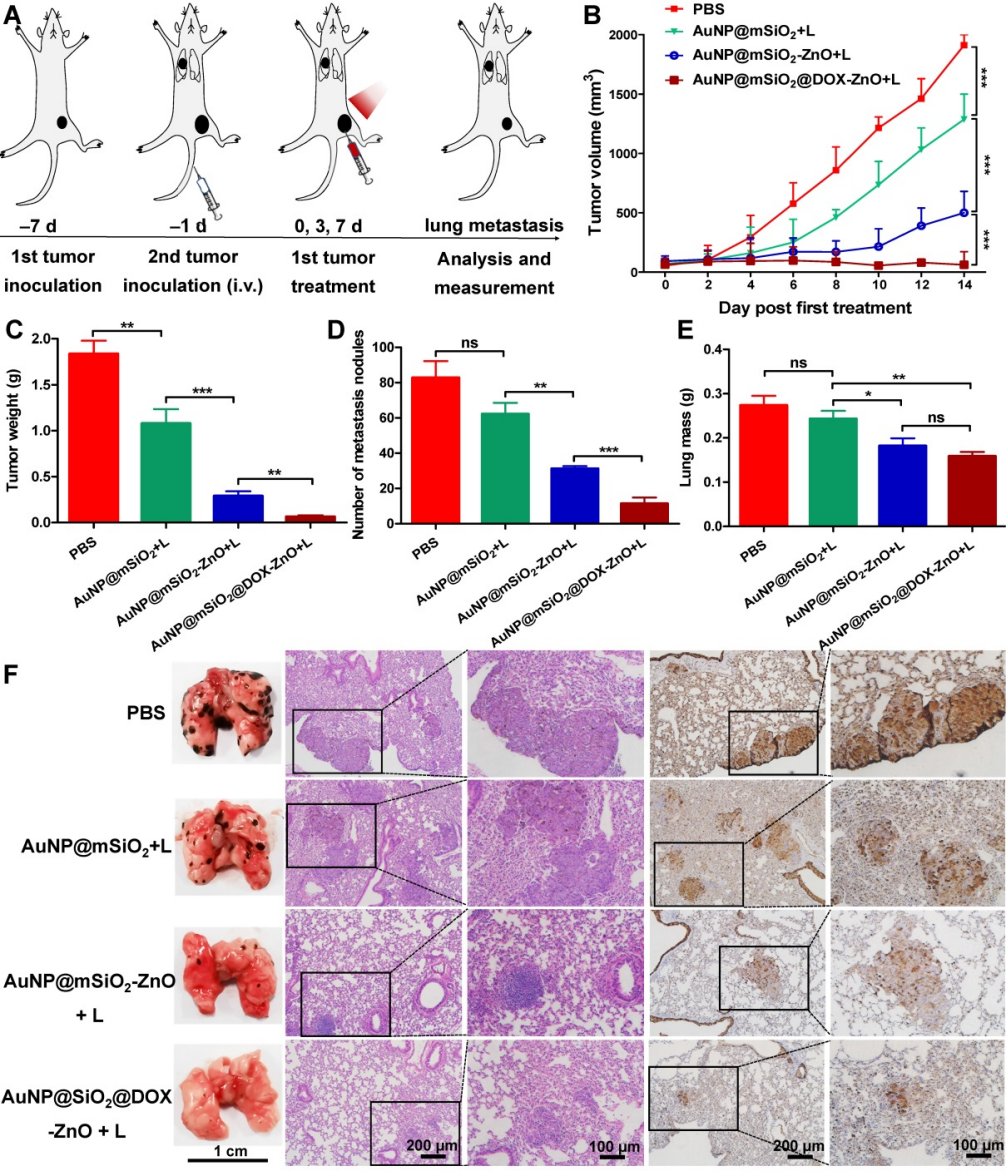
AuNP@mSiO_2_@DOX-ZnO prevents lung metastasis. (A) Schematic illustration exhibiting the design of animal experiments for studying lung metastasis. (B) Tumor volume of the primary tumors in each group with increasing time for 14 days after treatment. (C) All the tumors were harvested on day 14. The tumor weight of the primary tumors in each group was measured at the end of the experiment. (D, E) Numbers of metastatic nodules in the lungs and lung masse in each group after different treatments. (F) Representative lung photographs and histological examination of lung metastatic nodules with H&E and S100 staining. L refers to laser irradiation. Statistical significance was calculated by the *t*-test. The scale bars in the last images can be applied to the others in the same column. Data was presented as mean ± SD (n=5). The error bars represent the standard deviation. ns refers to not significant, *P < 0.05, **P < 0.01, ***P < 0.001.

As AuNP@mSiO_2_@DOX-ZnO may play a role in enhancing anti-tumor immunity, we wanted to investigate if this nanocomposite also could have potential for eliminating metastatic melanoma. Bilateral subcutaneous melanoma mice models were employed to determine whether the induced antitumor immune response from AuNP@mSiO_2_@DOX-ZnO could be effective against metastatic tumors. The design principle of animal experiments is shown in Figure 6A. We randomized B16/F10-injected mice into 5 groups and treated them with PBS, AuNP@mSiO_2_-ZnO, AuNP@mSiO_2_ + L, AuNP@mSiO_2_-ZnO + L, AuNP@mSiO_2_@DOX-ZnO + L, respectively. The primary tumors were injected with an intratumoral injection of these drugs every three days for a total of three injections. For mice with the primary tumors treated by AuNP@mSiO_2_-ZnO + L, their growth of secondary tumors was significantly delayed. The AuNP@mSiO_2_@DOX-ZnO + L group exhibited a greater inhibition in tumor growth. The groups treated with PBS, AuNP@mSiO_2_-ZnO, AuNP@mSiO_2_ + L did not show a significant suppressive effect in tumor growth or in the weight of secondary tumors. (Figure 6B-D and Figure S16). Additionally, we established melanoma mice models of lung metastases to further determine whether AuNP@mSiO_2_@DOX-ZnO could be effective against distant tumors. A similar trend was observed in the inhibition effect of AuNP@mSiO_2_@DOX-ZnO + L on lung metastasis. As seen in Figure 7 and Figure S17, the number of lung metastatic colonies was significantly reduced in the AuNP@mSiO_2_@DOX-ZnO + L group, followed by the AuNP@mSiO_2_-ZnO + L group. This data suggests that the ZnO-triggered combination strategy can significantly prevent the growth of tumors at distant sites.

**Figure 8:**
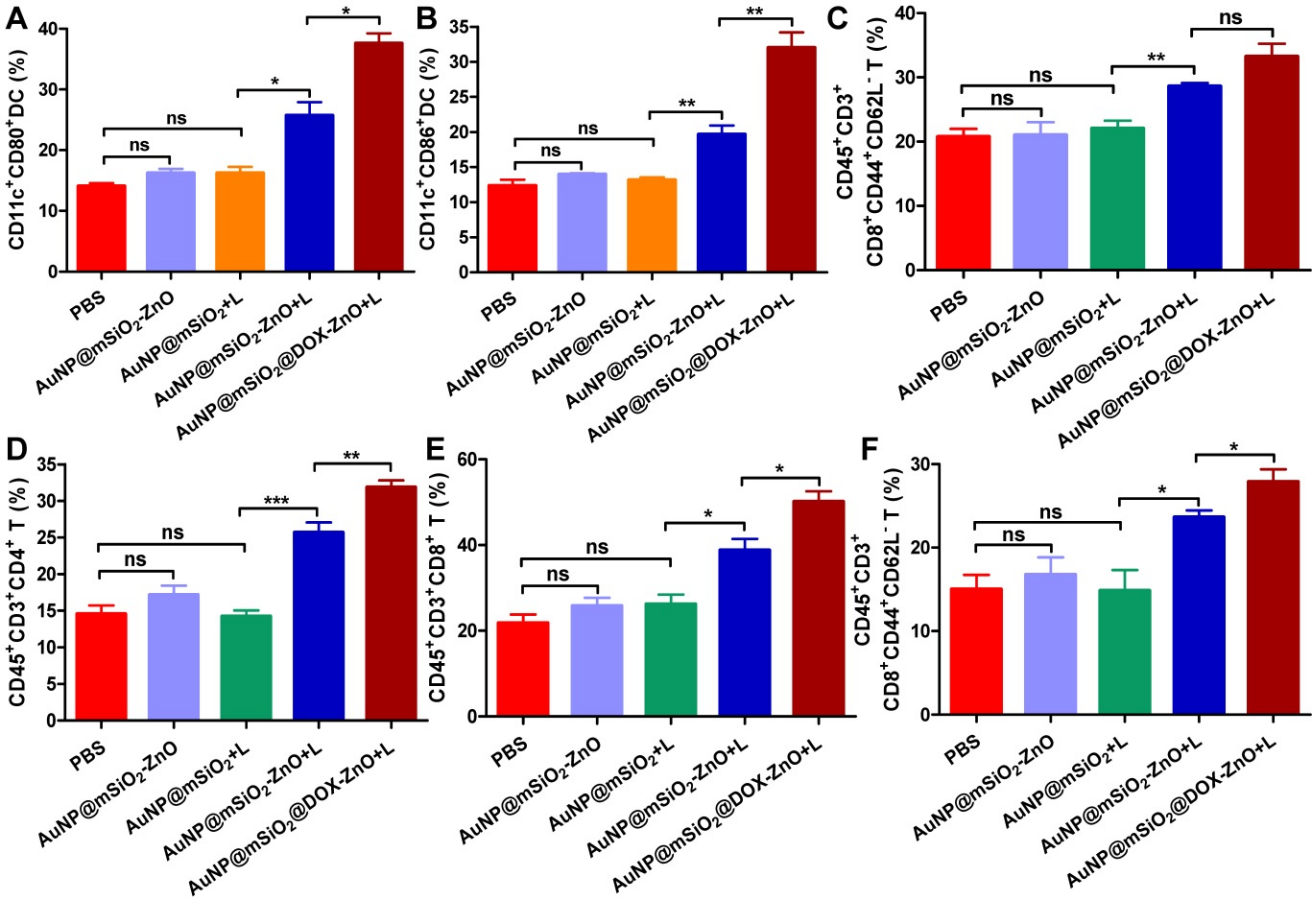
AuNP@mSiO_2_@DOX-ZnO-based combined therapy activated systemic anti-tumor immune responses. The immune cells in secondary melanoma tumors and their TDLNs were studied on day 14 after first treatment. (A-C) Flow cytometry analysis of the percentages of CD11c^+^CD80^+^ DC, CD11c^+^CD86^+^ DC and activated CD8^+^ T cells (PI^-^CD45^+^CD3^+^CD8^+^CD44^+^CD62L^-^) in TDLNs. (D-F) The distant tumors were harvested and the percentages of PI^-^CD45^+^CD3^+^CD4^+^ T cells, PI^-^CD45^+^CD3^+^CD8^+^ T cells and activated CD8^+^ T cells in total tumor cells were also determined by flow cytometry. L refers to laser irradiation. Statistical significance was calculated by the *t*-test. Data was presented as mean ± SD. The error bars represent the standard deviation. *P < 0.05, **P < 0.01, ***P < 0.001. TDLNs: tumor draining lymph nodes.

Moreover, to further identify the anti-tumor immune response triggered by AuNP@mSiO_2_@DOX-ZnO, immune cells in secondary melanoma tumors and their TDLNs were studied on day 14^th^ after the first treatment. Compared to groups of PBS, AuNP@mSiO_2_-ZnO and AuNP@mSiO_2_ + L, the percentages of CD11c^+^CD80^+^ DC and CD11c^+^CD86^+^ DC in TDLNs of AuNP@mSiO_2_-ZnO + L and AuNP@mSiO_2_@DOX-ZnO groups were significantly increased (Figure 8A-B). Also, the AuNP@mSiO_2_@DOX-ZnO + L group exhibited the highest percentages of activated CD8^+^CD44^+^CD62L^-^ T cells among all the treated groups (Figure 8C). Furthermore, compared to PBS, AuNP@mSiO_2_-ZnO and AuNP@mSiO_2_ + L, significantly increased proportions of infiltrating CD4^+^ T cells, CD8^+^ T cells and activated CD8^+^CD44^+^CD62L^-^ T cells were also observed in secondary tumors of the AuNP@mSiO_2_-ZnO + L and AuNP@mSiO_2_@DOX-ZnO + L groups (Figure 8D-F). Collectively, this data indicates that combined treatment with AuNP@mSiO_2_-based PTT and ZnO would significantly prevent the growth of distant tumors by triggering an anti-tumor immune response. Furthermore, AuNP@mSiO_2_-based PTT can only directly kill *in situ* tumors but can't inhibit metastatic tumors via activation of anti-tumor immunity. Although AuNP@mSiO_2_-ZnO can induce immunogenic cell death, it still cannot effectively inhibit tumor growth and metastasis. We postulated that the AuNP@mSiO_2_-based PTT would directly kill *in situ* tumor and further release tumor-associated antigens (TAAs), whereas ZnO acted as an adjuvant to incite TAA-specific anti-tumor immunity. Consistent with our findings, Shen, et al reported that the ZnO NPs could serve as an adjuvant to promote cellular and humoral immunity for brain tumor immunotherapy 23, and ZnO NPs could also affect the expression of CD44 in tumor stem cells, polarize macrophages towards to M1 phenotype and increase the secretion of IL-6 and TNF-α, exhibiting an adjuvant function 21. Nevertheless, our work is the first demonstration of the ability of ZnO NPs to inhibit melanoma metastasis by inducing an anti-tumor immune response when combined with photothermal therapy. Moreover, when combined with the cytotoxic and ICD-induced effect of DOX, the anti-tumor immune response is further enhanced, which could potentially lead to significant inhibition of melanoma metastases.

### Safety evaluation

During this period of treatment and observation, no significant body weight loss was observed (Figure S18). To further identify the potential side effects of AuNP@mSiO_2_-ZnO nanocomposites, normal C57 mice were intravenously injected with AuNP@mSiO_2_-ZnO at varying concentrations from 60 to 200 μg per mouse for 15 days. Then, routine blood analysis, serum biochemistries and H&E staining of major organs were performed. No significant difference was observed in routine hematologic and biochemical parameters between the AuNP@mSiO_2_-ZnO treatment and control groups at doses of 200 μg (Figure S19 and S20). On the other hand, compared with the control group, no detectable tissue damage or inflammation was observed in major organs from the mice injected with AuNP@mSiO_2_-ZnO (200 μg per mouse) (Figure S19C). These results suggested that AuNP@mSiO_2_-ZnO would be a promising nanoplatform with good safety profiles within a certain concentration range, providing a potential strategy for combined treatment of melanoma.

## Conclusions

**Scheme 1:**
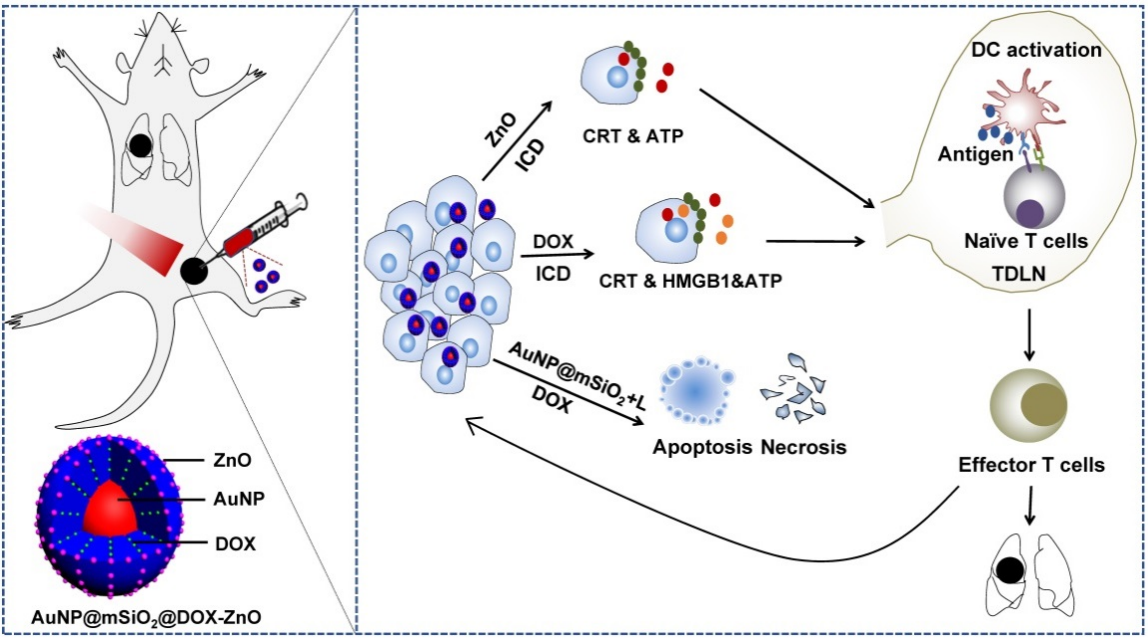
Schematic illustration showing the mechanism of ZnO-triggered combination therapy. ZnO NPs can not only guard pH-responsive targeted drug delivery but also induce ICD, which shows as adjuvant function. Also, the AuNP@mSiO_2_-based PTT can result in apoptosis and necrosis directly. DOX not only resulted in apoptosis and necrosis but also elicited ICD. The combination strategy promotes the maturation of DCs and presents tumor antigens to naïve T cells, further stimulating the infiltration of tumor-specific effector T cells into primary and metastatic tumors, preventing the progression and metastasis of melanoma. ICD represents Immunogenic Cell Death while L refers to laser irradiation.

In summary, we demonstrate the generation of a versatile and immunogenic nanocomposite, AuNP@mSiO_2_@DOX-ZnO, which was synthesized with a facile and efficient strategy and exhibited a high drug payload. Importantly, ZnO can not only guard pH-responsive targeted drug delivery, preferential phagocytic ability to melanoma cells but also induce an ICD effect, which can potentially trigger anti-tumor immunity. The combination strategy of AuNP@mSiO_2_@DOX-ZnO + L realized complementary advantages and induced an effective tumor-specific immune response, forcefully inhibited tumor growth and lung metastasis with lower exposer time and power intensity (schematically illustrated in Scheme 1). The complex mechanisms underlying this adjuvant behavior and synergic strategy displayed by ZnO NPs in cancer treatment need further investigation. Nevertheless, the results presented here encourage the wider pursuit of combined nano-based immunotherapy and present a promising combination strategy with promising potential in the clinical application of melanoma therapy.

## Supplementary Material

Supplementary figures and tables.

## Abbreviations

Au@SiO_2_: mesoporous silica-coated gold nanoparticles
ATP: adenosine triphosphate
CTAB: cetyl trimethyl ammonium bromide
CLSM: confocal laser scanning microscope
DAMPs: damage-associated molecular patterns
DC: dendritic cell
DOX: doxorubicin
H&E: hematoxylin and eosin
HMGB1: high-mobility group box 1
HIFs: hypoxia-inducible factors
ICD: immunogenic cell death
IHC: staining and immunohistochemistry
MFI: mean fluorescence intensity
NPs: nanoparticles
PBS: phosphate buffer solution
PTT: photothermal therapy
ROS: reactive oxygen species
SEM: scanning electron microscopy
TAAs: tumor-associated antigens
TAMs: tumor-associated macrophages
TDLNs: tumor draining lymph nodes
TEM: transmission electron microscope

## References

[1] Michielin O, Hoeller C (2015). Gaining momentum: new options and opportunities for the treatment of advanced melanoma. Cancer Treat Rev.

[2] Long GV, Atkinson V, Lo S, Sandhu S, Guminski AD, Brown MP (2018). Combination nivolumab and ipilimumab or nivolumab alone in melanoma brain metastases: a multicentre randomised phase 2 study. Lancet Oncol.

[3] Zhang Z, Wang J, Nie X, Wen T, Ji Y, Wu X (2014). Near infrared laser-induced targeted cancer therapy using thermoresponsive polymer encapsulated gold nanorods. J Am Chem Soc.

[4] Wang C, Xu L, Liang C, Xiang J, Peng R, Liu Z (2014). Immunological responses triggered by photothermal therapy with carbon nanotubes in combination with anti-CTLA-4 therapy to inhibit cancer metastasis. Adv Mater.

[5] Zou L, Wang H, He B, Zeng L, Tan T, Cao H (2016). Current approaches of photothermal therapy in treating cancer metastasis with nanotherapeutics. Theranostics.

[6] Wang X, Lv F, Li T, Han Y, Yi Z, Liu M (2017). Electrospun micropatterned nanocomposites incorporated with Cu2S nanoflowers for skin tumor therapy and wound healing. ACS Nano.

[7] Wang L, Yuan Y, Lin S, Huang J, Dai J, Jiang Q (2016). Photothermo-chemotherapy of cancer employing drug leakage-free gold nanoshells. Biomaterials.

[8] Song J, Yang X, Jacobson O, Lin L, Huang P, Niu G (2015). Sequential drug release and enhanced photothermal and photoacoustic effect of hybrid reduced graphene oxide-loaded ultrasmall gold nanorod vesicles for cancer therapy. ACS Nano.

[9] Zhang Z, Wang L, Wang J, Jiang X, Li X, Hu Z (2012). Mesoporous silica-coated gold nanorods as a light-mediated multifunctional theranostic platform for cancer treatment. Adv Mater.

[10] Ramya AN, Joseph MM, Maniganda S, Karunakaran V, T (2017). T. S, Maiti KK. Emergence of gold-mesoporous silica hybrid nanotheranostics: dox-encoded, folate targeted chemotherapy with modulation of SERS fingerprinting for apoptosis toward tumor eradication. Small.

[11] Huo X, Dai C, Tian D, Li S, Li X (2015). Au@SiO2 core-shell structure involved with methotrexate: fabrication, biodegradation process and bioassay explore. Int J Pharm.

[12] Feng Q, Yang X, Hao Y, Wang N, Feng X, Hou L (2019). Cancer cell membrane-biomimetic nanoplatform for enhanced sonodynamic therapy on breast cancer via autophagy regulation strategy. ACS Appl Mater Interfaces.

[13] Zhang Z, Liu C, Bai J, Wu C, Xiao Y, Li Y (2015). Silver nanoparticle gated, mesoporous silica coated gold nanorods (AuNR@MS@AgNPs): low premature release and multifunctional cancer theranostic platform. ACS Appl Mater Interfaces.

[14] Vankayala R, Lin CC, Kalluru P, Chiang CS, Hwang KC (2014). Gold nanoshells-mediated bimodal photodynamic and photothermal cancer treatment using ultra-low doses of near infra-red light. Biomaterials.

[15] Xu X, Li Y, Li H, Liu R, Sheng M, He B (2014). Smart nanovehicles based on pH-triggered disassembly of supramolecular peptide-amphiphiles for efficient intracellular drug delivery. Small.

[16] Song Y, Li Y, Xu Q, Liu Z (2016). Mesoporous silica nanoparticles for stimuli-responsive controlled drug delivery: advances, challenges, and outlook. Int J Nanomedicine.

[17] Rim HP, Min KH, Lee HJ, Jeong SY, Lee SC (2011). pH-tunable calcium phosphate covered mesoporous silica nanocontainers for intracellular controlled release of guest drugs. Angew Chem Int Ed.

[18] Niedermayer S, Weiss V, Herrmann A, Schmidt A, Datz S, Müller K (2015). Multifunctional polymer-capped mesoporous silica nanoparticles for pH-responsive targeted drug delivery. Nanoscale.

[19] Muhammad F, Guo M, Qi W, Sun F, Wang A, Guo Y (2011). pH-triggered controlled drug release from mesoporous silica nanoparticles via intracelluar dissolution of ZnO nanolids. J Am Chem Soc.

[20] Ye DX, Ma YY, Zhao W, Cao HM, Kong JL, Xiong HM (2016). ZnO-based nanoplatforms for labeling and treatment of mouse tumors without detectable toxic side effects. ACS Nano.

[21] Martínez-Carmona M, Gun'ko Y, Vallet-Regí M (2018). ZnO nanostructures for drug delivery and theranostic applications. Nanomaterials (Basel).

[22] Duan X, Chan C, Lin W (2019). Nanoparticle-mediated immunogenic cell death enables and potentiates cancer immunotherapy. Angew Chem Int Ed.

[23] Shen Q, Yang J, Liu R, Liu LY, Zhang J, Shen S (2019). Hybrid 'clusterbombs' as multifunctional nanoplatforms potentiate brain tumor immunotherapy. Mater Horiz.

[24] Chen JC, Zhang RY, Han L, Tu B, Zhao DY (2013). One-pot synthesis of thermally stable gold@mesoporous silica core-shell nanospheres with catalytic activity. Nano Res.

[25] Cai X, Luo Y, Zhang W, Du D, Lin Y (2016). pH-sensitive ZnO quantum dots-doxorubicin nanoparticles for lung cancer targeted drug delivery. ACS Appl Mater Interfaces.

[26] Kodiha M, Mahboubi H, Maysinger D, Stochaj U (2016). Gold nanoparticles impinge on nucleoli and the stress response in MCF7 breast cancer cells. Nanobiomedicine (Rij).

[27] Jiang J, Pi J, Cai J (2018). The advancing of zinc oxide nanoparticles for biomedical applications. Bioinorg Chem Appl.

[28] Rasmussen JW, Martinez E, Louka P, Wingett DG (2010). Zinc oxide nanoparticles for selective destruction of tumor cells and potential for drug delivery applications. Expert Opin Drug Deliv.

[29] Sharma P, Jang NY, Lee JW, Park BC, Kim YK, Cho NH (2019). Application of ZnO-based nanocomposites for vaccines and cancer immunotherapy. Pharmaceutics.

[30] Lin LS, Wang JF, Song J (2019). Cooperation of endogenous and exogenous reactive oxygen species induced by zinc peroxide nanoparticles to enhance oxidative stress-based cancer therapy. Theranostics.

[31] Gabrilovich DI, Ostrand-Rosenberg S, Bronte V (2012). Coordinated regulation of myeloid cells by tumours. Nat Rev Immunol.

[32] Noy R, Pollard JW (2014). Tumor-associated macrophages: from mechanisms to therapy. Immunity.

[33] George S, Pokhrel S, Xia T, Gilbert B, Ji ZX, Schowalter M (2010). Use of a rapid cytotoxicity screening approach to engineer a safer zinc oxide nanoparticle through iron doping. ACS Nano.

[34] Zhang HJ, Shan YF, Dong LJ (2014). A comparison of TiO2 and ZnO nanoparticles as photosensitizers in photodynamic therapy for cancer. J Biomed Nanotechnol.

[35] Chu KF, Dupuy DE (2014). Thermal ablation of tumours: biological mechanisms and advances in therapy. Nat Rev Cancer.

[36] Chen Q, Xu L, Liang C, Wang C, Peng R, Liu Z (2016). Photothermal therapy with immune-adjuvant nanoparticles together with checkpoint blockade for effective cancer immunotherapy. Nat Commun.

[37] Guo J, Rahme K, He Y, Li LL, Holmes JD, O'Driscoll CM (2017). Gold nanoparticles enlighten the future of cancer theranostics. Int J Nanomedicine.

[38] Wang Q, Ju X, Wang J, Fan Y, Ren M, Zhang H (2018). Immunogenic cell death in anticancer chemotherapy and its impact on clinical studies. Cancer Lett.

[39] Sweeney EE, Cano-Mejia J, Fernandes R (2018). Photothermal therapy generates a thermal window of immunogenic cell death in neuroblastoma. Small.

[40] Tanaka M, Kataoka H, Yano S, Sawada T, Akashi H, Inoue M (2016). Immunogenic cell death due to a new photodynamic therapy (PDT) with glycoconjugated chlorin (G-chlorin). Oncotarget.

[41] Li W, Yang J, Luo L, Jiang M, Qin B, Yin H (2019). Targeting photodynamic and photothermal therapy to the endoplasmic reticulum enhances immunogenic cancer cell death. Nat Commun.

[42] Huang Z, Wei G, Zeng Z, Huang Y, Huang L, Shen Y (2019). Enhanced cancer therapy through synergetic photodynamic/immune checkpoint blockade mediated by a liposomal conjugate comprised of porphyrin and IDO inhibitor. Theranostics.

[43] Sethuraman SN, Singh MP, Patil G, Li S, Fiering S, Hoopes PG (2020). Novel calreticulin-nanoparticle in combination with focused ultrasound induces immunogenic cell death in melanoma to enhance antitumor immunity. Theranostics.

[44] Dai X, Meng J, Deng S, Zhang L, Wan C, Lu L (2020). Targeting CAMKII to reprogram tumor-associated macrophages and inhibit tumor cells for cancer immunotherapy with an injectable hybrid peptide hydrogel. Theranostics.

[45] Lim S, Park J, Shim MK, Um W, Yoon HY, Ryu JH (2019). Recent advances and challenges of repurposing nanoparticle-based drug delivery systems to enhance cancer immunotherapy. Theranostics.

[46] Kroemer G, Galluzzi L, Kepp O, Zitvogel L (2013). Immunogenic cell death in cancer therapy. Annu Rev Immunol.

[47] Garg AD (2016). Agostinis P. Editorial: immunogenic cell death in cancer: from benchside research to bedside reality. Front Immunol.

[48] Zhao X, Yang K, Zhao R, Ji T, Wang X, Yang X (2016). Inducing enhanced immunogenic cell death with nanocarrier-based drug delivery systems for pancreatic cancer therapy. Biomaterials.

[49] Huang FY, Lei J, Sun Y, Yan F, Chen B, Zhang L (2018). Induction of enhanced immunogenic cell death through ultrasound-controlled release of doxorubicin by liposome-microbubble complexes. Oncoimmunology.

[50] Galluzzi L, BuquéA, Kepp O, Zitvogel L, Kroemer G (2017). Immunogenic cell death in cancer and infectious disease. Nat Rev Immunol.

[51] Xu J, Su Y, Xu A, Fan F, Mu S, Chen L (2019). miR-221/222-mediated inhibition of autophagy promotes dexamethasone resistance in multiple myeloma. Mol Ther.

[52] Inoue H, Tani K (2014). Multimodal immunogenic cancer cell death as a consequence of anticancer cytotoxic treatments. Cell Death Differ.

