# Supplementary material for: ZnO-based multifunctional nanocomposites to inhibit progression and metastasis of melanoma by eliciting antitumor immunity via immunogenic cell death
Source: Theranostics. 2020 Sep 14;10(24):11197–214. doi: 10.7150/thno.44920 (PMC7532661; doi:10.7150/thno.44920)
Supplement: Supplementary file 1 — Supplementary figures and tables. [file thnov10p11197s1.pdf]

Supporting Information

ZnO-based multifunctional nanocomposites to inhibit progression and metastasis of melanoma by eliciting antitumor immunity via immunogenic cell death

Yamin Zhang,[1] Chen Guo,[2] Liping Liu,[2] Jian Xu,[3] Hao Jiang,[2] Danqi Li,[1] Jiajia Lan,[1] Jun Li,[4] Jing Yang,[1] Qiming Tu,[5] Xiaoyan Sun,[6] Mahin Alamgir,[7] Xiang Chen,[8] Guanxin Shen,[9] Jintao Zhu,[2] Juan Tao[*,1]

[1] Department of Dermatology, Union Hospital, Tongji Medical College, Huazhong University of Science and Technology (HUST), Wuhan 430022, China

[2] Key Laboratory of Material Chemistry for Energy Conversion and Storage, Ministry of Education, School of Chemistry and Chemical Engineering, HUST, Wuhan 430074, China

[3] Department of Hematology, Union Hospital, Tongji Medical College, HUST, Wuhan 430022, China

[4] Department of Dermatology, The Central Hospital of Wuhan, Tongji Medical College, HUST, Wuhan 430022, China

[5] Clinical Laboratory, Union Hospital, Tongji Medical College, HUST, Wuhan 430022, China

[6] Department of Dermatology, Tongji Hospital, Tongji Medical College, Huazhong University of Science and Technology (HUST), Wuhan 430022, China

[7] Department of Dermatology, Rutgers-RWJMS, Somerset, New Jersey, USA

[8] Department of Dermatology, XiangYa Hospital, Central South University, Changsha 410008, China

[9] Department of Immunology, Tongji Medical College, HUST, Wuhan 430022, China

* Corresponding author. *E-mail*: tjhappy@126.com (J. Tao)

Contents

Supplementary experimental methods (Page S4-S5)

Figure S1. Characterization and photothermal properties of AuNP@mSiO_2-ZnO. (Page S5-S6)

Figure S2. Compositional Characterization of AuNP@mSiO_2-ZnO. (Page S6-7)

Figure S3. Representative CLSM images of AuNP@mSiO_2@DOX-ZnO nanocomposites uptaken by B16/F10 melanoma cells *in vitro*. (Page S7-8)

Figure S4. Cellular uptake and internalization of AuNP@mSiO_2 or AuNP@mSiO_2-ZnO nanocomposite *in vivo*. (Page S8)

Figure S5. The endocytosis ability of B16/F10 cells and TAMs for AuNP@mSiO_2-ZnO-Cy5 *in vivo* and cell viability of AuNP@mSiO_2-ZnO or ZnO NPs for B16/F10 cells. (Page S9)

Figure S6. The photothermal effect of AuNP@mSiO2-ZnO on B16F10 melanoma cells. *in vitro*. (Page S10)

Figure S7. The *in vivo* fluorescence images of CY5-labeled AuNP@mSiO_2-ZnO after intratumoral administration. (Page S11)

Figure S8. The Kaplan−Meier survival curves of in-situ tumor growth inhibition experiment. (Page S11)

Figure S9. The anti-tumor effect of AuNP@mSiO_2-ZnO with various time and power intensity of laser irradiation. (Page S12)

Figure S10. Representative flow cytometry plots showing antitumor immune responses induced by AuNP@mSiO_2@DOX-ZnO with laser irradiation. (Page S13)

Figure S11. Representative CLSM images of $CD3^+CD8^+$ T cells in tumor treated by AuNP@mSiO_2@DOX-ZnO with laser irradiation. (Page S14)

Figure S12. Cell apoptosis and necrosis of B16/F10 melanoma cells induced by AuNP@mSiO_2@DOX-ZnO. (Page S15)

Figure S13. The morphological characteristics of B16/F10 cells treated with AuNP@mSiO_2-ZnO + L. (Page

S16)

Figure S14. ZnO NPs induced autophagic cell death via ROS production. (Page S17)

Figure S15. AuNP@mSiO_2-ZnO induced B16/F10 tumor cells to release DAMP via ROS *in vitro*. (Page S18)

Figure S16. The photographs, tumor volumes and weight of primary tumors in bilateral subcutaneous melanoma mice model. (Page S19)

Figure S17. The photographs of the primary tumors and lungs in lung metastasis mice model. (Page S20)

Figure S18. Body weight of tumor-bearing mice from different groups. (Page S21)

Figure S19. *In vivo* biosafety assessment of AuNP@mSiO_2-ZnO. (Page S22)

Figure S20. Blood routine analysis of normal mice after treatment. (Page S23)

Table S1. Characteristics of the core-shell AuNP@mSiO_2 and AuNP@mSiO_2-ZnO from BET analysis. (Page S24)

Supplementary experimental methods

Materials.

Cetyltrimethylammonium bromide (CTAB, 99.0%), amino propyl tri ethoxy silane (APTES, 99.0%) and 2, 5-Furandione, dihydro-3-[3-(tri ethoxy silyl) propyl] (TESPA, 99%) were purchased from Sigma-Aldrich. Zn $(CH_3COO)_2 \cdot 2H_2O$ was obtained from Aladdin. Formaldehyde solution (37.0%), zincacetate dihydrate and Tetraethyl orthosilicate (TEOS) were purchased from sinopharm chemical reagent Co., Ltd. Deionized water (Millipore Milli-Q grade) with resistivity of 18.0 MΩ was used in all the experiments. All chemicals were of analytical grade and were used as received without further purification.

Photothermal performance of AuNP@mSiO$_2$-ZnO.

The AuNP@mSiO$_2$-ZnO aqueous solutions at various concentrations were irradiated with 655 nm laser with varied power densities and time. The solution temperatures were detected by thermocouples (SC-GG-K-30-36, Omega, USA) and PBS was used as control.

In-vivo distribution.

Mice model of subcutaneous melanoma was generated as previously described. PBS or Cy5-labeled AuNP@mSiO$_2$-ZnO nanocomposites were intratumorally injected to tumor-bearing mice with a dose of 60 μg per mice. Animals were sacrificed (n = 5) at 1 h, 3 h, 6 h and 12 h after injection and the fluorescence images were obtained by a live small animal optical imaging system (IVIS Lumina XR; Caliper).

Immunofluorescence.

Tumors as mentioned above were collected and the frozen tissue sections were prepared. Then, the sections were air-dried, fixed in acetone and blocked in 1% bovine serum albumin for 30 minutes at 37 °C. After washing with PBS, the sections were incubated with diluting fluorescent primary antibodies of CD3 (145-2C11) and CD8 (53-6.7) for another 30 minutes at 37 °C, stained with DAPI, and observed under confocal laser scanning microscope (Leica TCSNT1, Wetzlar Germany).

In-vivo biosafety analysis.

Healthy female C57 mice were intravenously injection with AuNP@mSiO$_2$-ZnO at dosages from 65 to 200 μg (60 μg, 100 μg and 200 μg) per mice (n = 5). 15 days later, all the mice were sacrificed and blood samples were collected for blood routine analysis (Sysmex 1800i, Japan) and blood biochemistry test (Hitachi 7600, Japan). In addition, the major organs, including heart, liver, spleen, lung and kidney, were harvested and fixed with 4% paraformaldehyde for H&E staining.

Supplementary Figures:

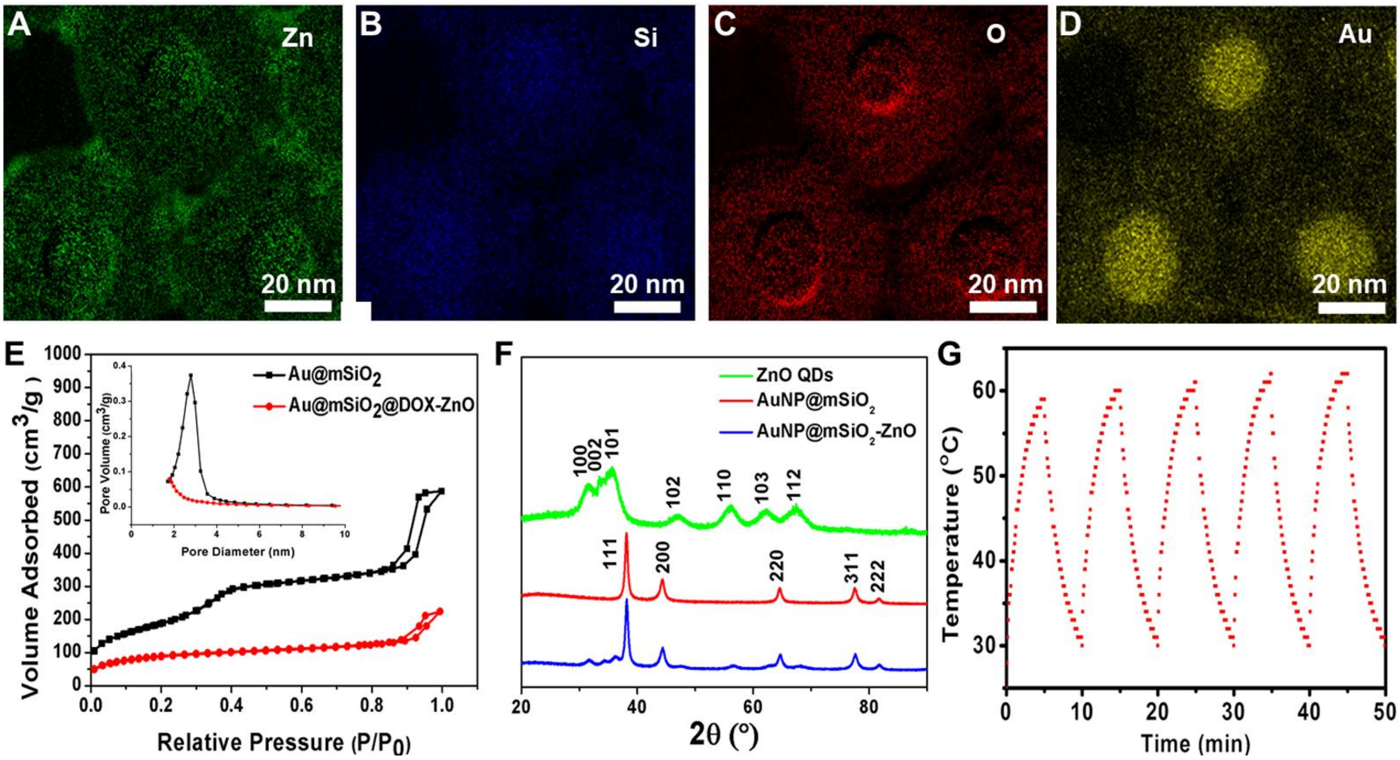

Figure S1. Characterization and photothermal properties of AuNP@mSiO$_2$-ZnO. (A-D) STEM-EDX mapping for Zn, Si, O and Au to demonstrate the presence of all the four elements in AuNP@mSiO$_2$-ZnO nanocomposites. (E) N_2 adsorption/desorption isotherms of AuNP@mSiO$_2$ and AuNP@mSiO$_2$-ZnO nanoparticles, displayed typical IV isotherm according to the IUPAC classification, showing the characteristic of mesopores. The pore size distribution (inset) exhibited a sharp peak centered at the mean value of 2.8 nm,

indicating a uniform mesopore. The BET surface area and total pore volume were calculated to be 686 m^2/g and 0.9 cm^3/g, respectively. (F) The wide-angle X-ray diffraction (XRD) pattern indicated the successful fabrication of AuNP@mSiO_2-ZnO, compared with those of ZnO QDs and AuNP@mSiO_2. (G) The photothermal heating curves of AuNP@mSiO_2-ZnO solution irradiated with 2.0 W/cm^2 NIR for 5 min and turned off for 5 min for 5 cycles to examine the photothermal stability of AuNP@mSiO_2-ZnO, and this process was repeated for continuous five cycles. We observed that the temperature of AuNP@mSiO_2-ZnO did not decrease significantly, which indicated that the photothermal stability of AuNP@mSiO_2-ZnO was high.

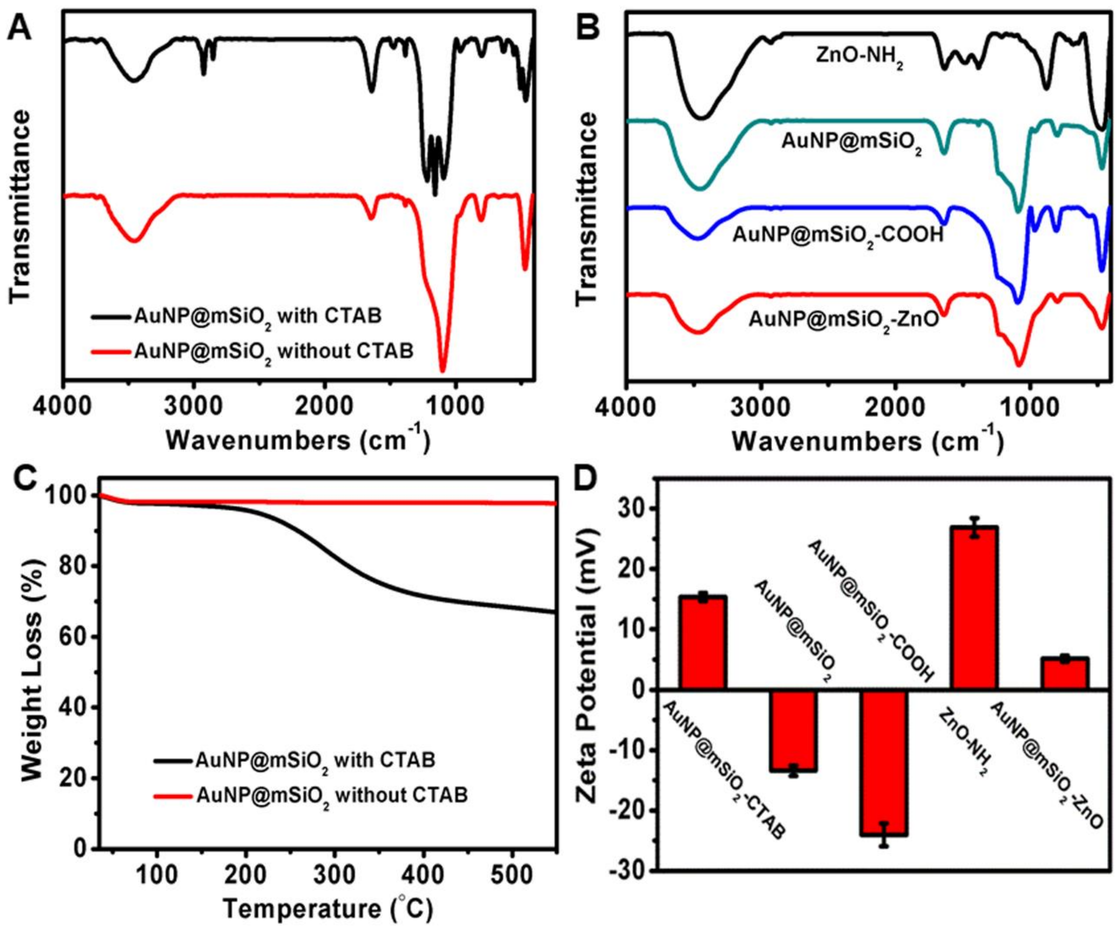

Figure S2. (A) FTIR spectra of AuNP@SiO_2 before and after CTAB in the mesoporous channels was removed using ethanol solution of HCl through ion exchange. The absence of the asymmetric stretching vibrations of alkyl groups displayed at 2800-2950 cm^{-1} indicated the removement of CTAB. (B) FTIR spectra of ZnO-NH_2, AuNP@mSiO_2, AuNP@mSiO_2-COOH and AuNP@mSiO_2-ZnO. The presence of −N−H bending

vibration and the asymmetric stretching vibrations of alkyl groups at 1567 cm^{-1} and 2954 cm^{-1}, respectively, revealed the successful modification of ZnO QDs with APTES. In the case of AuNP@mSiO_2-ZnO, the formation of amide bond between AuNP@mSiO_2-COOH and ZnO-NH_2 could be demonstrated by the presence of the peaks at 1572 cm^{-1} and 1665 cm^{-1}. (C) TGA curves of AuNP@SiO_2 with and without CTAB revealed the removement of CTAB. (D) Zeta potentials of AuNP@mSiO_2, AuNP@mSiO_2-COOH, ZnO QD and AuNP@mSiO_2-ZnO. Initially, zeta potential of AuNP@mSiO_2 changed from 15.5 ± 0.2 mV to −13.6 ± 0.3 mV with the removement of CTAB. Then, due to the presence of carboxylic acid group derived from TESPA, the zeta potential of AuNP@mSiO_2-COOH raised to −24.6 ± 0.8 mV. In the case of ZnO QD, the modified APTES with amine groups resulted in the positive charges and the zeta potential of ZnO-NH_2 was 26.8 ± 0.5 mV. The approximate neutral charge of AuNP@mSiO_2-ZnO could be ascribed to the formation of amide bond between AuNP@mSiO_2-COOH and ZnO-NH_2.

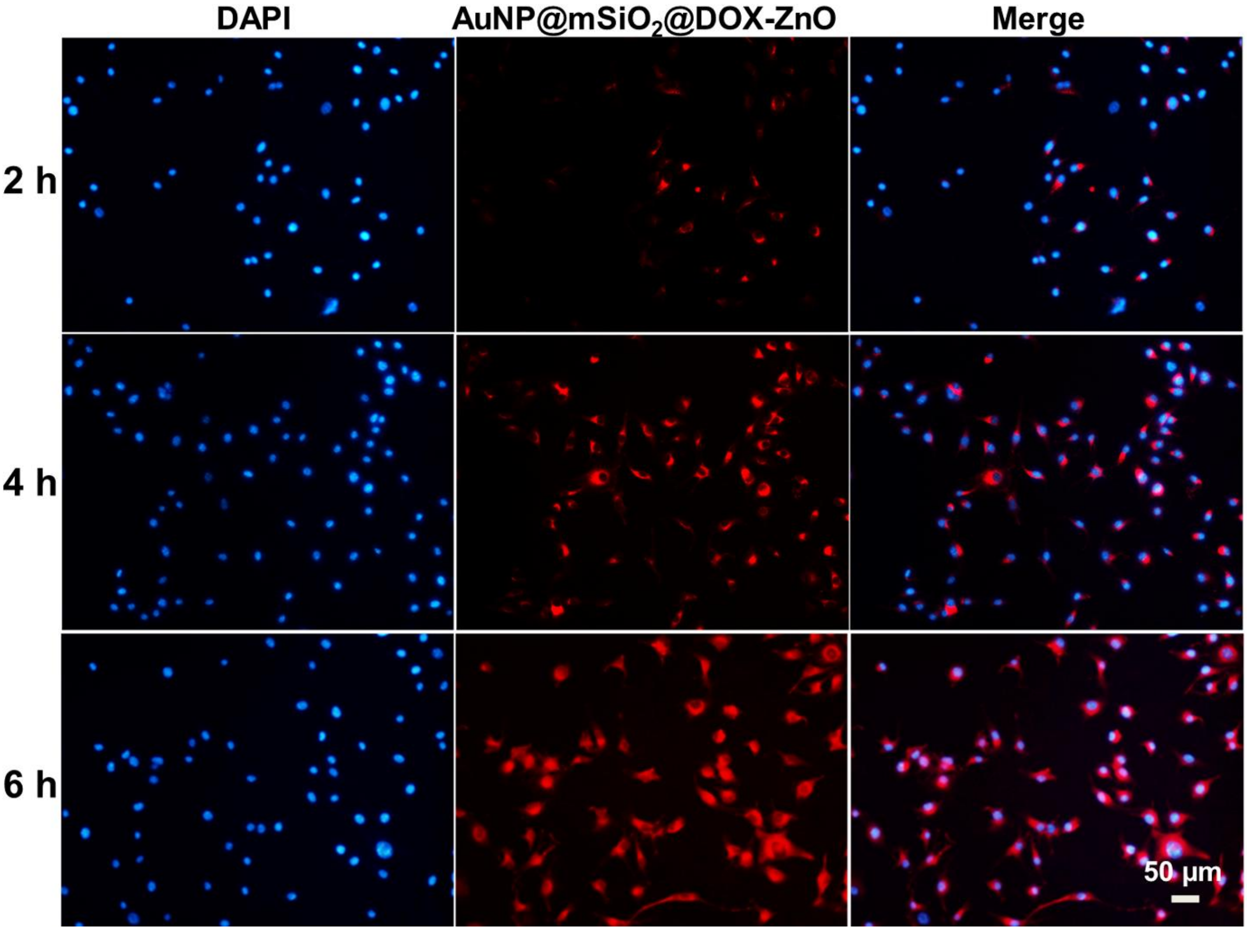

Figure S3. Representative CLSM images of AuNP@mSiO$_2$@DOX-ZnO nanocomposites uptaken by B16/F10 melanoma cells *in vitro*. The cells were incubated with AuNP@mSiO$_2$@DOX-ZnO (red) for 2 h, 4 h and 6 h, respectively. Cell nucleus were stained with DAPI (blue). The scale bar can be applied to all the other images and these experiments were performed in triplicate.

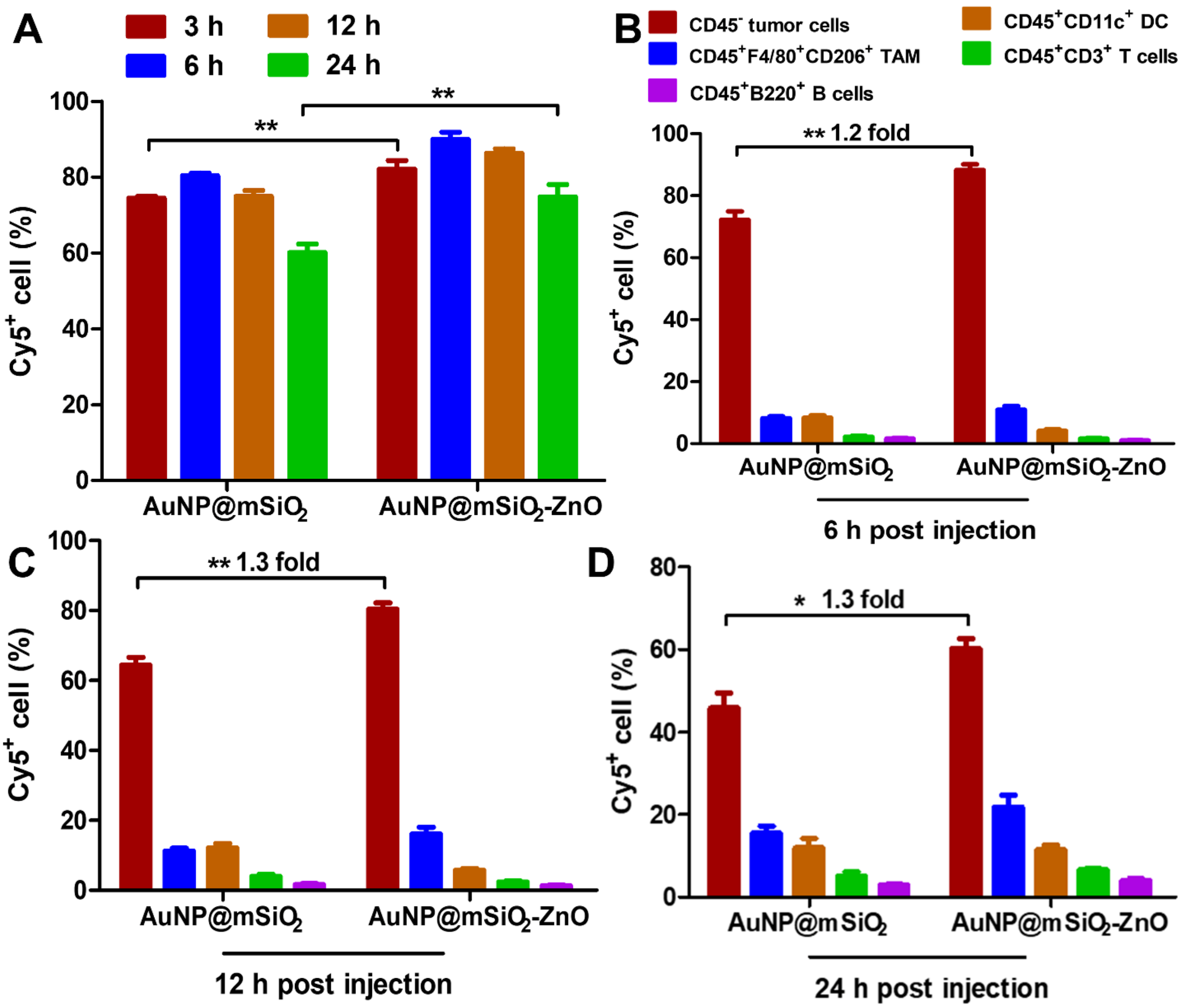

Figure S4. Cellular uptake and internalization of AuNP@mSiO$_2$ or AuNP@mSiO$_2$-ZnO nanocomposite *in vivo*. (A) The proportion of total cells which have phagocytosed AuNP@mSiO$_2$-ZnO-Cy5 and AuNP@mSiO$_2$-Cy5 at different times after intratumor injection. (B-D) The proportions of B16/F10 melanoma cells, TAM, DC, T and B cells that internalized AuNP@mSiO$_2$-ZnO-Cy5 or AuNP@mSiO$_2$-Cy5 were analyzed for virous times after intratumor injection. Results are representative of at least three independent experiments. Data was given as mean ± SD. Statistical significance was calculated by the t-test. **P* < 0.05, ***P* < 0.01.

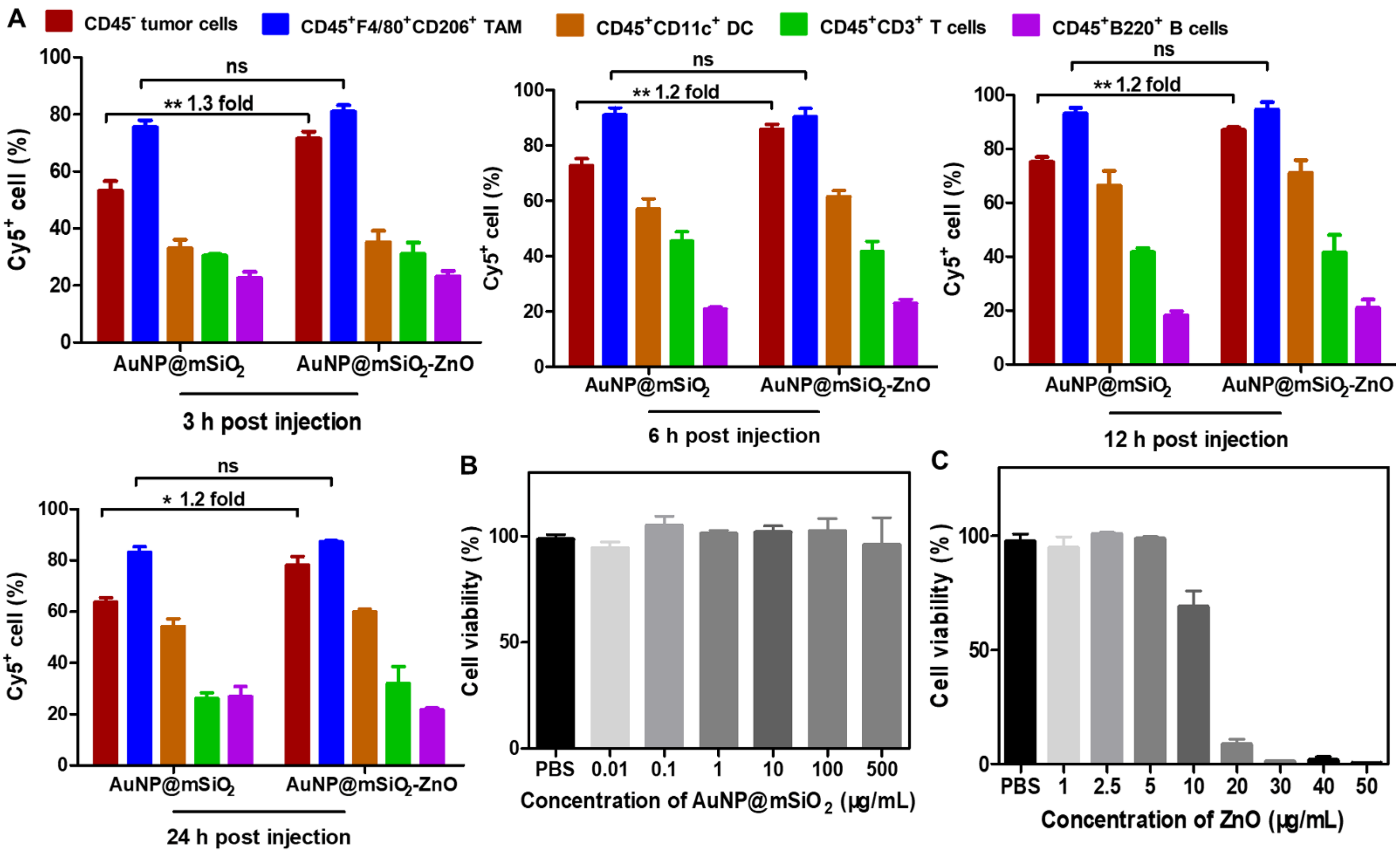

Figure S5. (A) Evaluation the endocytosis ability of AuNP@mSiO$_2$-ZnO-Cy5 *in vivo*. B16 melanoma cells, TAMs, DC, T cells and B cells were gated and the proportions of different cell types that internalized AuNP@mSiO$_2$-Cy5 or AuNP@mSiO$_2$-ZnO-Cy5 were analyzed. (B, C) Cell viability of B16/F10 cells treated with AuNP@mSiO$_2$-ZnO or ZnO with various concentrations for 24 h. Results are representative of at least three independent experiments. Data was given as mean ± SD. Statistical significance was calculated by the t-test. *P < 0.05, ***P < 0.001, ns, not significant.

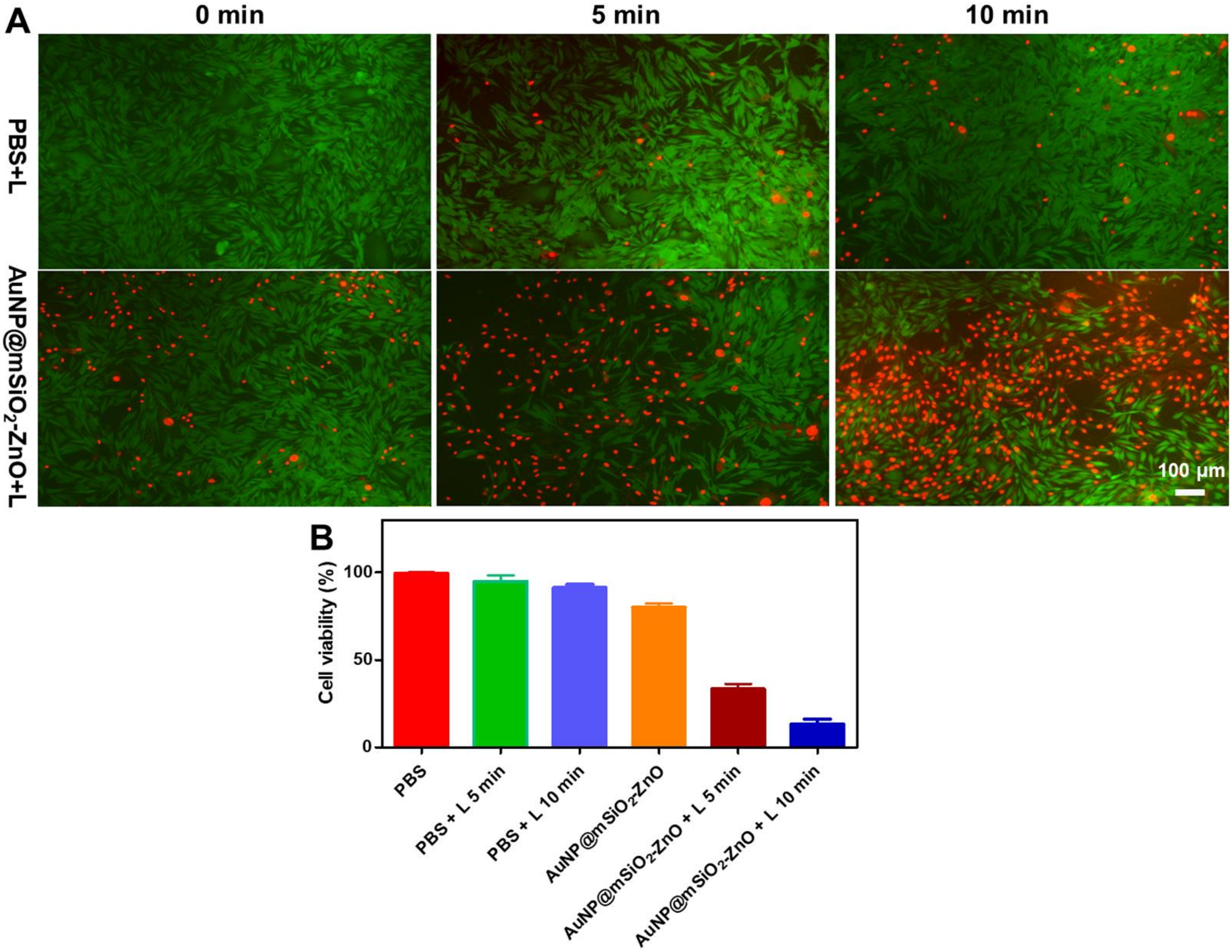

Figure S6. The photothermal effect of AuNP@mSiO_2-ZnO on B16F10 melanoma cells *in vitro*. (A) Representative live (green) / dead (red) cell staining images of B16F10 melanoma cells treated by AuNP@mSiO_2-ZnO with different irradiation duration (655 nm, 1.0 W/cm^2) *in vitro*. The scale bar in the last image can be applied to the other images. (B) B16/F10 cells were treated with AuNP@mSiO_2-ZnO or AuNP@mSiO_2-ZnO with laser irradiation for 5 or 10 min and the cell viability was detected by CCK8.

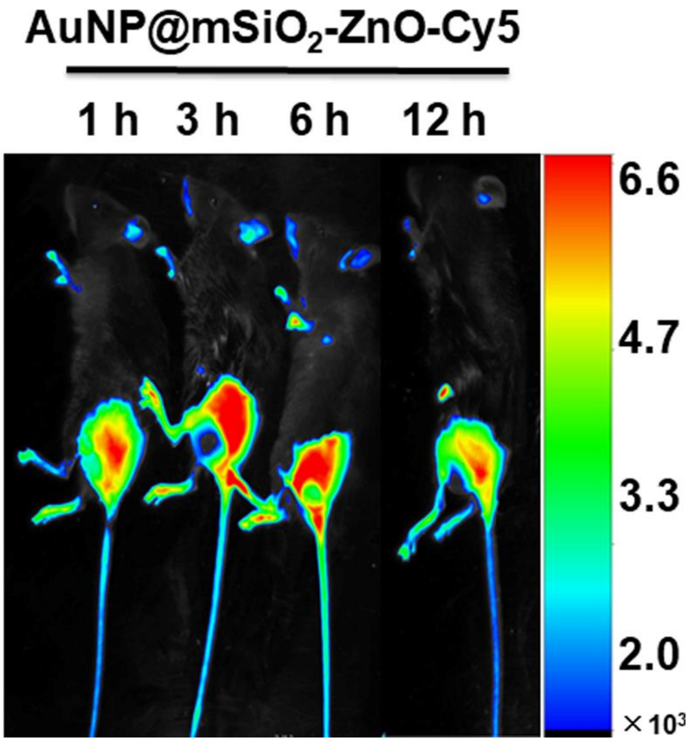

Figure S7. The fluorescence images of CY5-labeled AuNP@mSiO_2-ZnO at 1, 3, 6, and 12 h after intratumoral administration. Results are representative of at least three independent experiments.

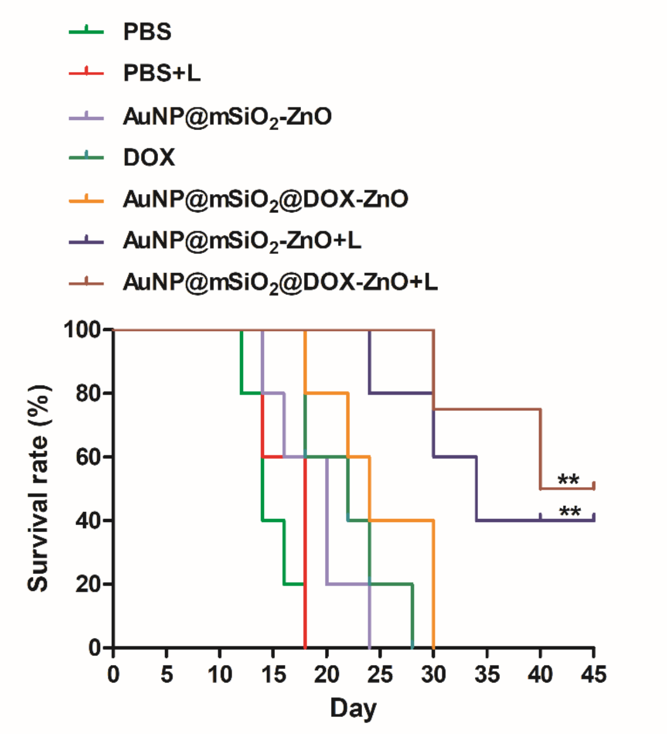

Figure S8. The Kaplan−Meier survival curves of in-situ tumor growth inhibition experiment (n = 5). L refers to laser irradiation. Comparisons of survival curves were made using the long-rank test. **$P < 0.01$.

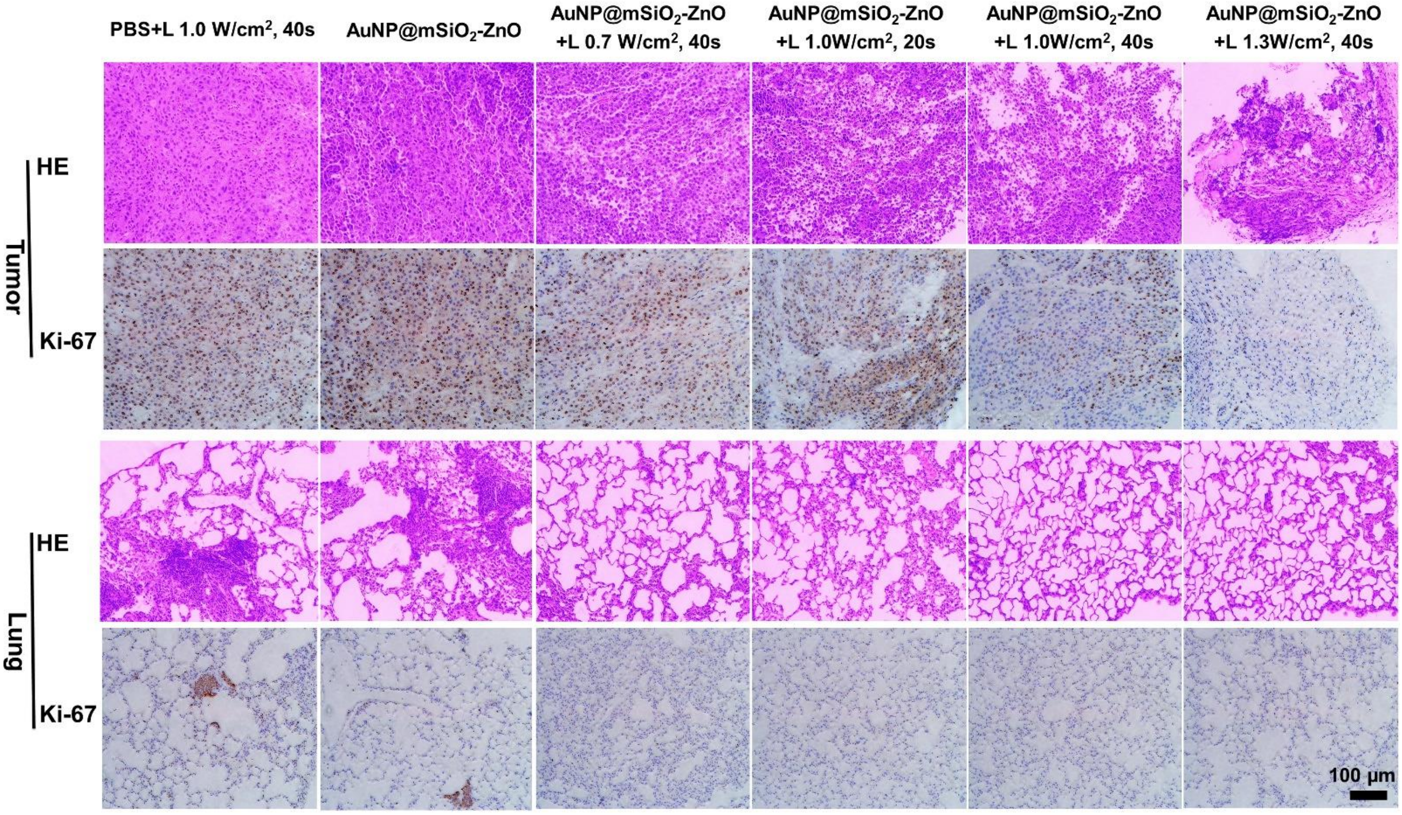

Figure S9. The anti-tumor effect of AuNP@mSiO_2-ZnO with various time and power intensity of laser irradiation. Tumor-bearing mice were treated every three days for a total of three injections. 16 days after, all the tumors and lungs were collected, sectioned and stained with hematoxylin and eosin (H&E) and Ki-67 (n = 5). Images are representative of 5 fields. The scale bars can be applied to all the other images.

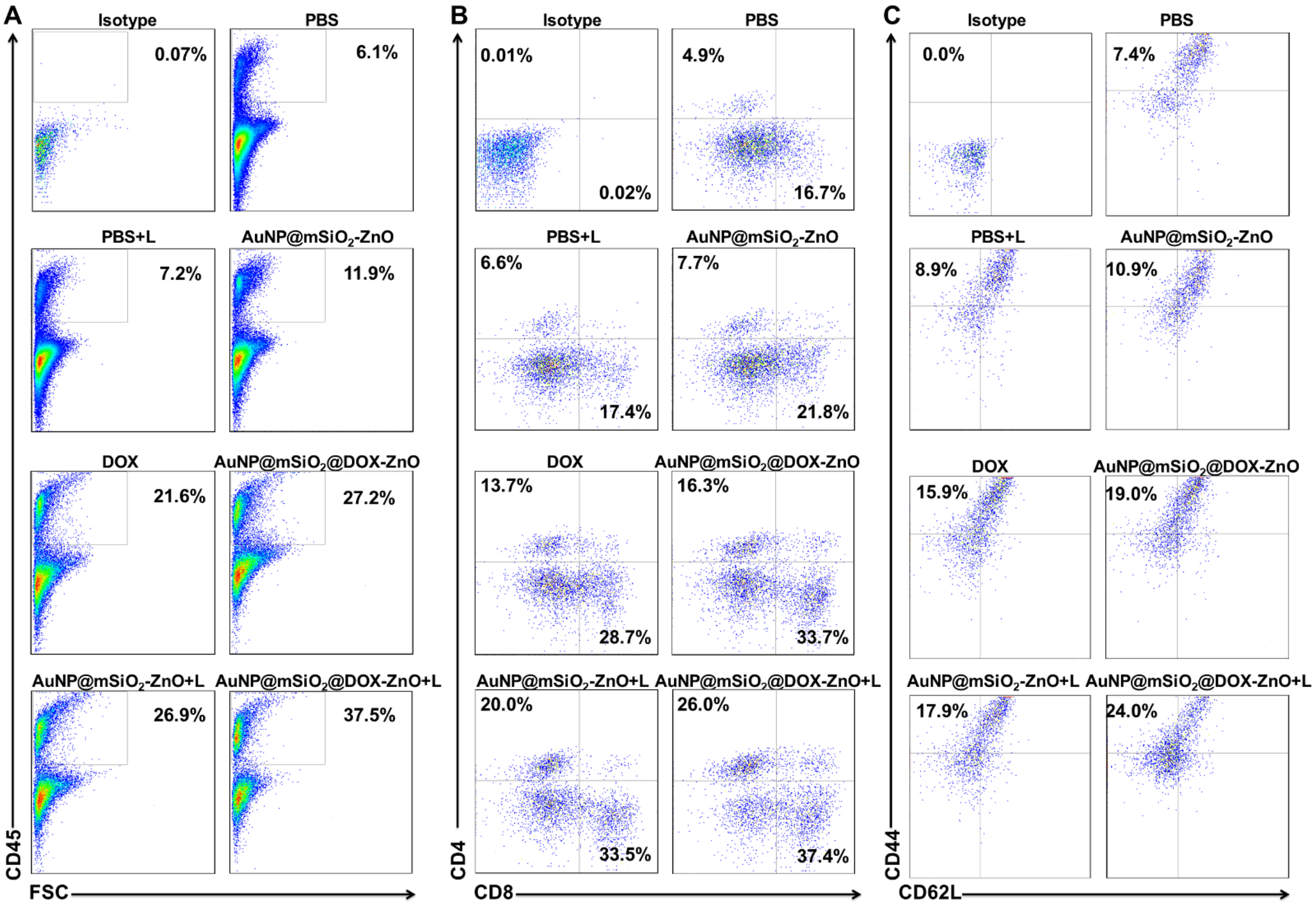

Figure S10. AuNP@mSiO2@DOX-ZnO with laser irradiation induced systematic antitumor immune responses. Representative flow cytometry plots showing the percentages of (A) $CD45^+$ leukocytes ($CD45^+PI^-$), (B) $CD4^+$ T cells ($CD45^+CD3^+CD4^+PI^-$)/$CD8^+$ T cells ($CD45^+CD3^+CD8^+PI^-$) and (C) activated $CD8^+$ T cells ($CD45^+CD3^+CD8^+$ $CD44^+CD62L^-PI^-$). Results are representative of at least three independent experiments.

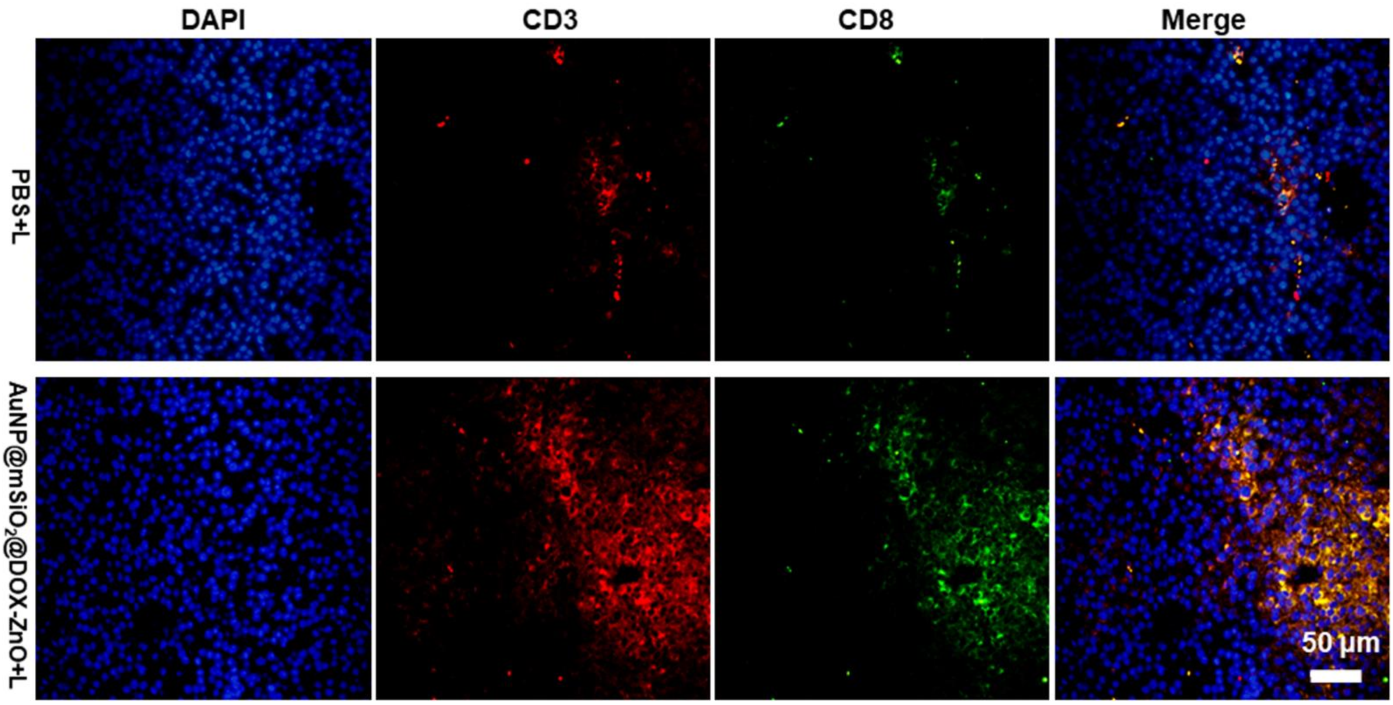

Figure S11. The tumor-bearing mice were treated as indicated and all the tumors were collected, sectioned and stained with DAPI (blue), CD3 (red) and CD8 (green). Representative CLSM images were showed after immunofluorescence staining (n = 5). Images are representative of 5 fields. The scale bars can be applied to all the other images.

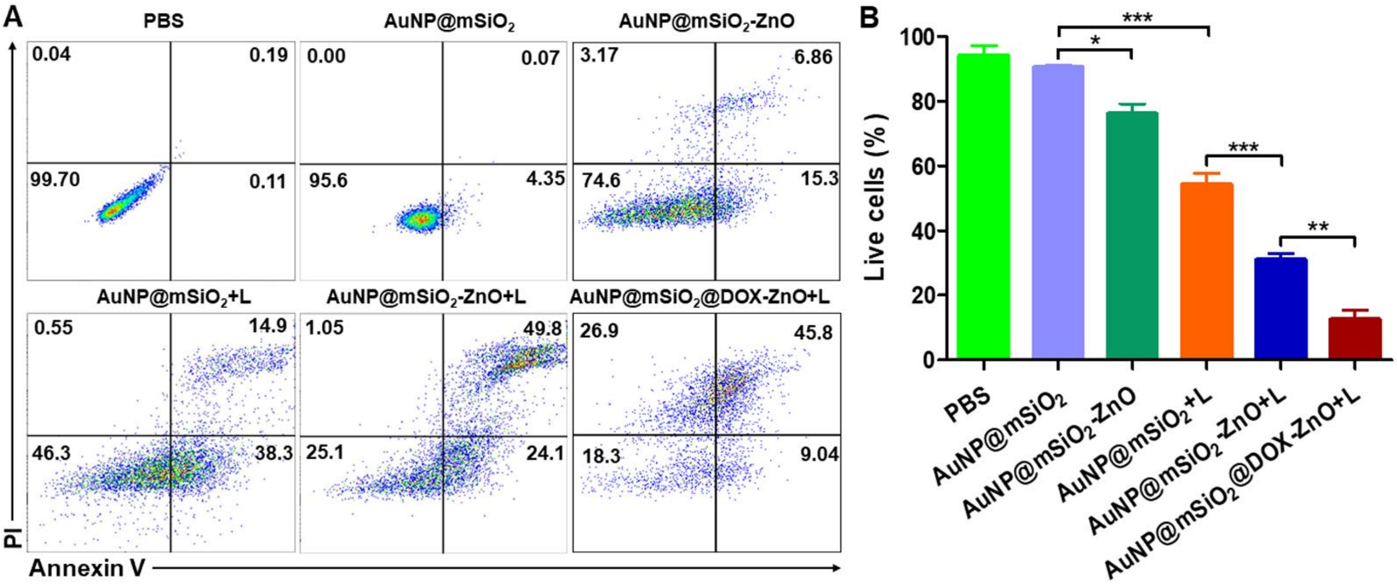

Figure S12. Flow cytometric analysis of cell apoptosis and necrosis of B16/F10 melanoma cells after treatment as indicated for 24 h by staining with Annexin V-FITC and PI. Live cells were also counted. Results are representative of at least three independent experiments. Data were given as mean ± SD. Statistical significance was calculated by the t-test. Data was presented as mean ± SD. **P* < 0.05, ***P* < 0.01, ****P* < 0.001.

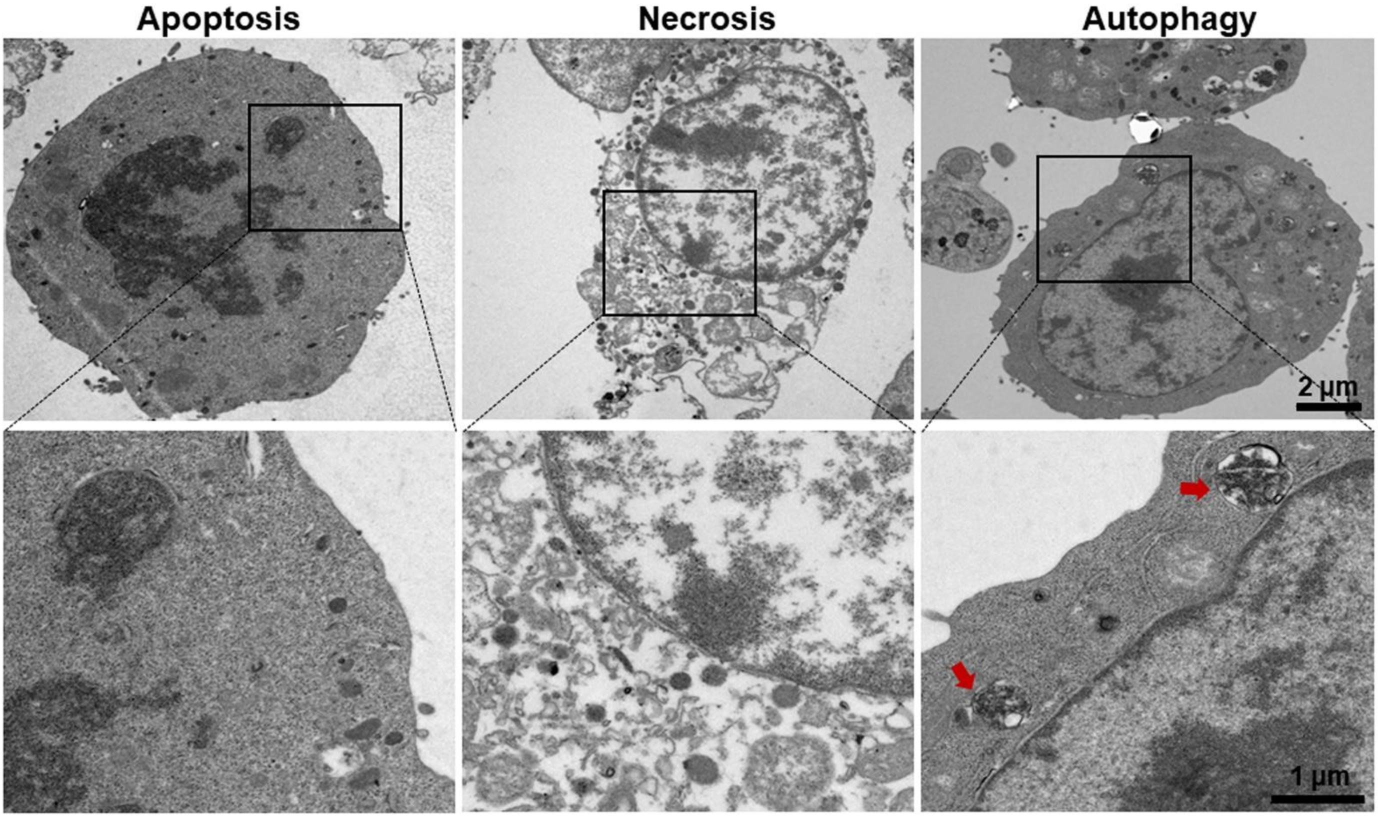

Figure S13. B16F10 melanoma cells were treated with AuNP@mSiO_2-ZnO + L (1.0 W/cm^2, 5 min) for 12 h and the morphological characteristics were observed by transmission electron microscopy. The autophagosomes or autolysosomes were represented by red arrows. The scale bars in the last images apply to the others in the same line. Results are representative of at least three independent experiments and images are representative of 5 fields.

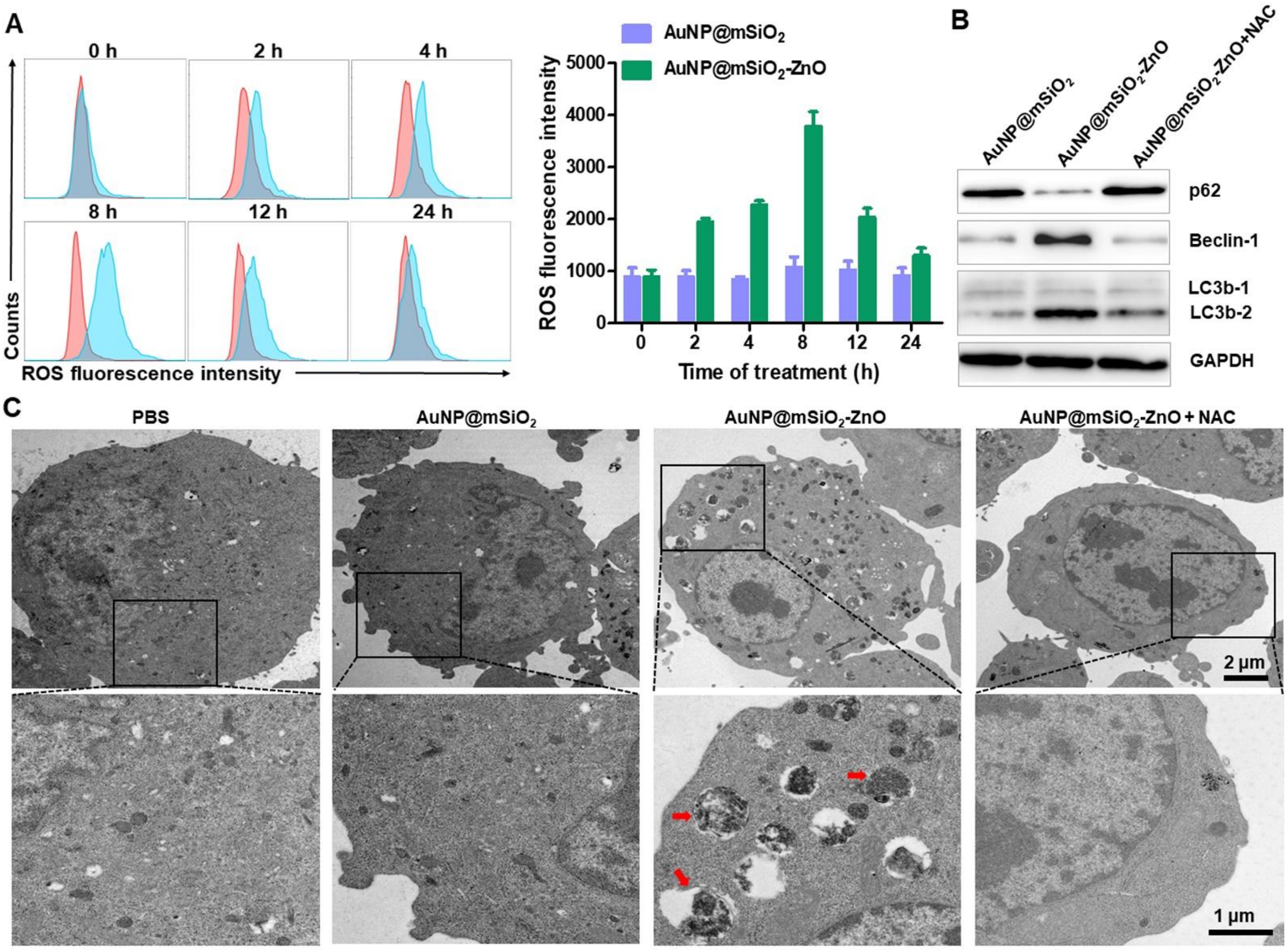

Figure S14. ZnO induced autophagic cell death via ROS production. (A) Effects of AuNP@mSiO_2 and AuNP@mSiO_2-ZnO on ROS level in B16/F10 melanoma cells for varying incubated times. (B) The B16/F10 melanoma cells were treated as indicated for 48 h, and the expressions of p62, Beclin-1 and LC3b were then detected by Western blots. (C) The autophagosomes or autolysosomes in AuNP@mSiO_2-ZnO group were observed in B16/F10 melanoma cells (red arrows). The scale bar in the last images applies to the others in the same line. Results are representative of at least three independent experiments. Data was presented as mean ± SD. NAC: oxidative stress inhibitor Nacetyl L-cysteine.

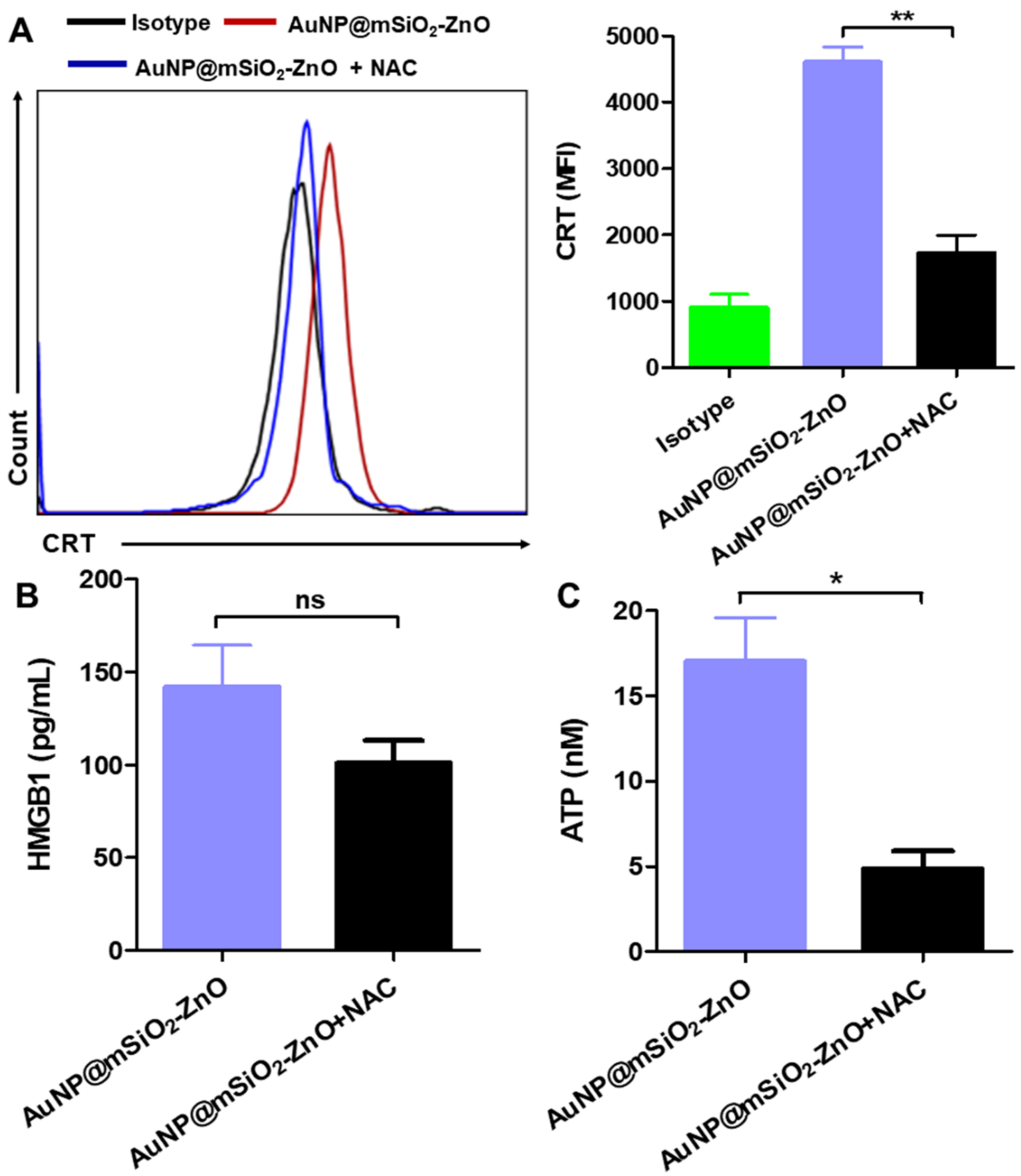

Figure S15. AuNP@mSiO_2-ZnO induced B16/F10 tumor cells to release DAMP via ROS *in vitro*. (A) Flow cytometric analysis of the CRT exposure on the surface of B16/F10 melanoma cells after treatment as indicated for 6 h. (B, C) HMGB1 and ATP secretion in culture supernatants after treatment for 72 h and 48 h, respectively. Results are representative of at least three independent experiments. Data was presented as mean ± SD. Statistical significance was calculated by the t-test. *P < 0.05, **P < 0.01, ns, not significant. NAC: oxidative stress inhibitor Nacetyl L-cysteine.

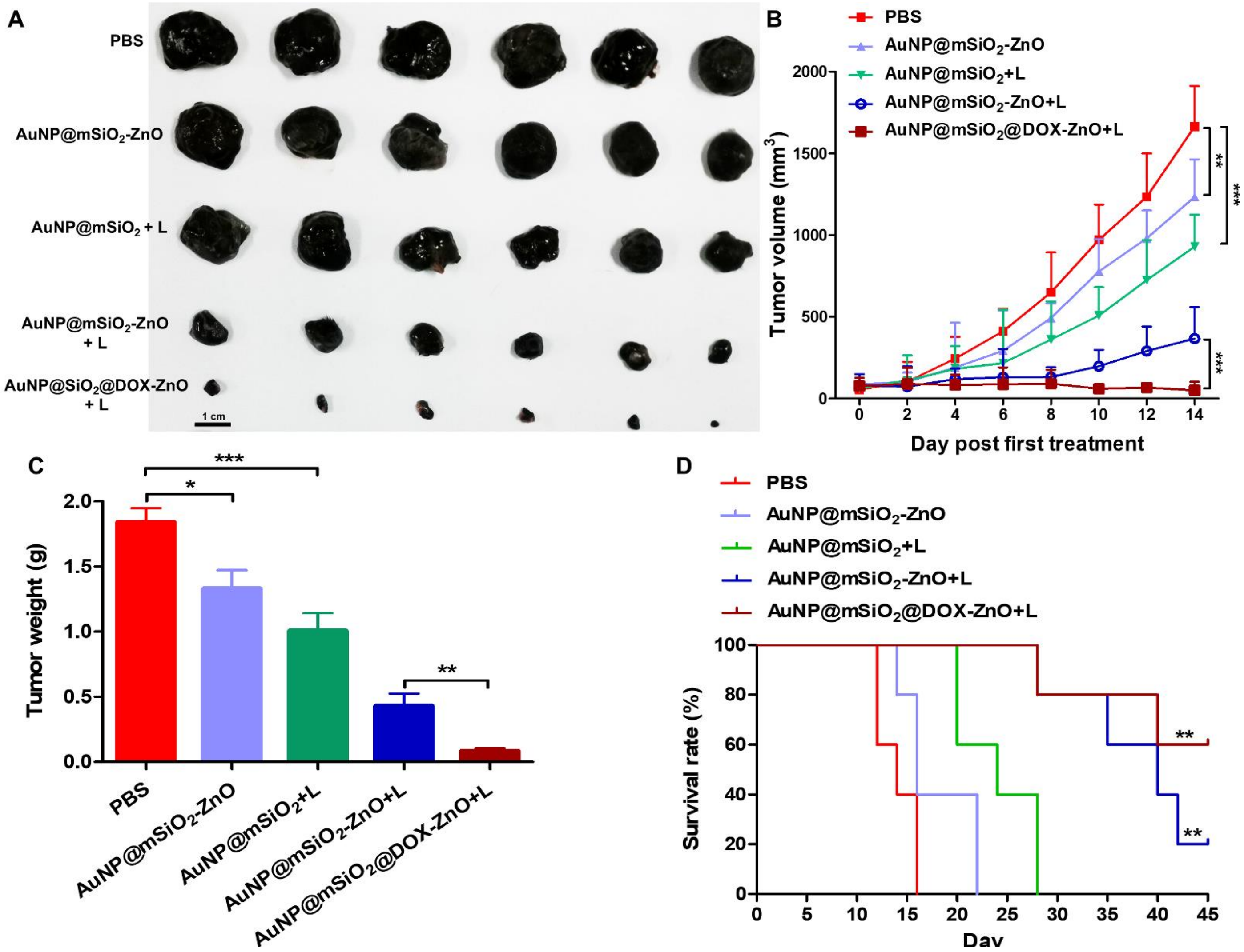

Figure S16. Bilateral subcutaneous melanoma mice model was established. The photographs (A), tumor volumes (B) and tumor weight (C) of the primary tumors in each group were measured at the end of experiment (n=6). (D) Kaplan−Meier survival curves. There were five mice per group. Statistical significance with two groups was calculated by the t-test. Comparisons of survival curves were made using the long-rank test. Data was presented as mean ± SD. *$P < 0.05$, **$P < 0.01$, ***$P < 0.001$.

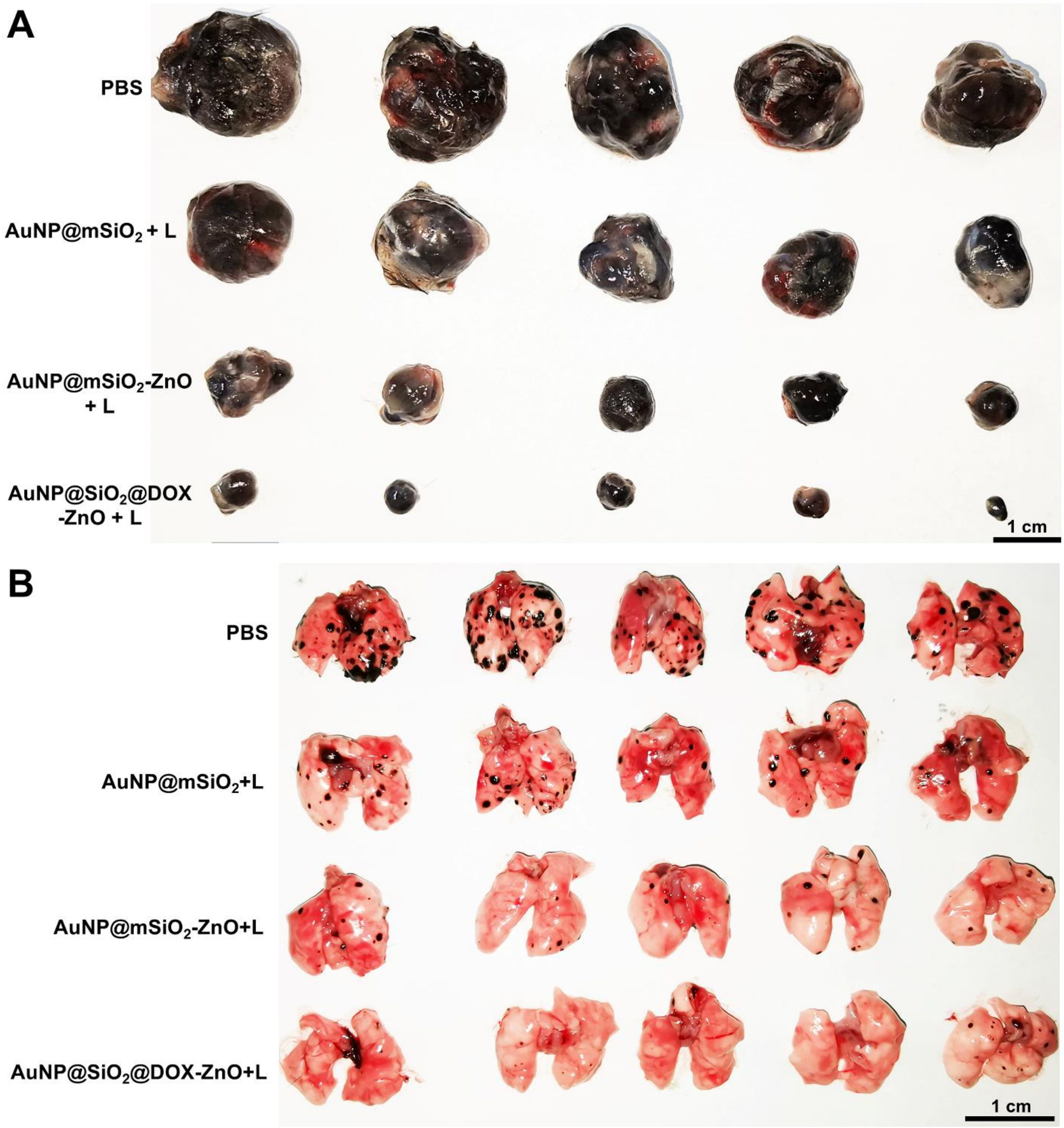

Figure S17. B16/F10 tumor cells were inoculated on the right flank of each mouse. A week later, a second tumor was i.v. injection of the same mouse as an artificial mimic of lung metastasis. In the following week, the 1st tumor of all the groups received intratumoral treatments every three days for a total of three times. The photographs of the primary tumors (A) and lungs (B) in each group were measured at the end of experiment.

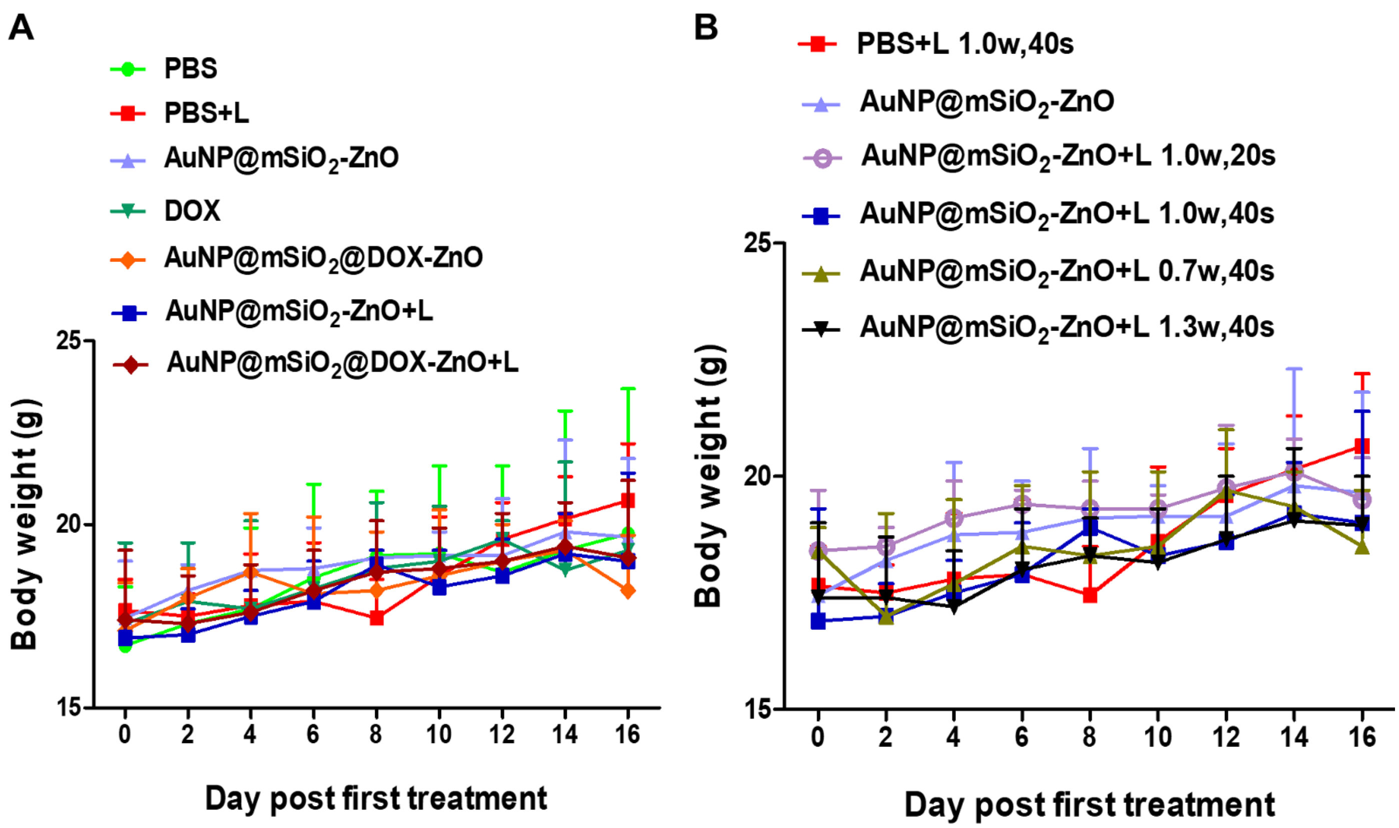

Figure S18. Body weight of tumor-bearing mice receiving treatments as indicated. Data was presented as mean ± SD (n=5).

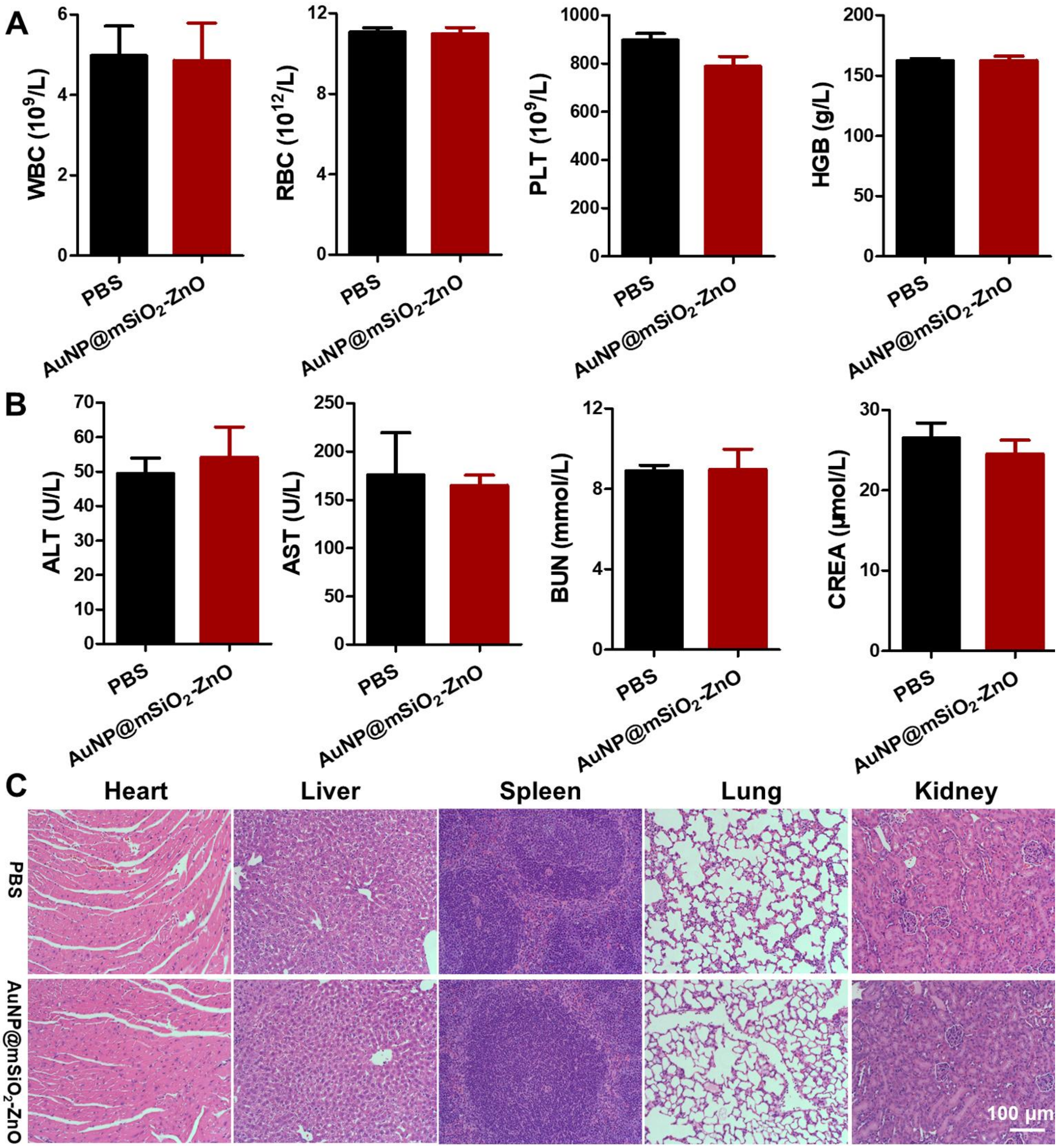

Figure S19. *In vivo* biosafety assessment of AuNP@mSiO$_2$-ZnO. Normal mice were intravenously injection with AuNP@mSiO2-ZnO at dosage of 200 μg per mice (n=5). 15 days later, the mice were sacrificed and blood samples and major organs were harvested. (A) Blood routine analysis: RBCs, WBCs, platelets PLT and HGB. (B) Blood biochemistry test: ALT, AST, BUN and CREA. (C) Representative H&E staining images of major organs (heart, liver, spleen, lung and kidney). The scale bar in the last image applies to the others. Data was presented as mean ± SD (n=5).

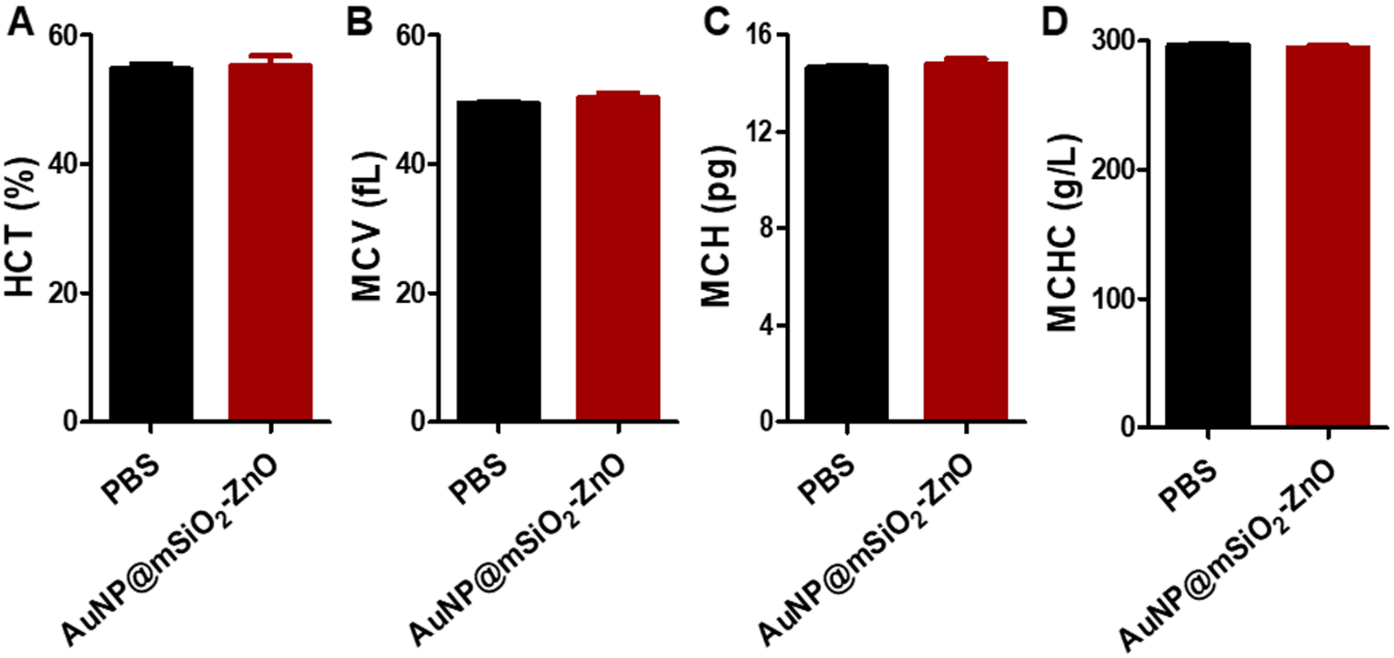

Figure S20. Blood routine analysis of normal mice after treatment with AuNP@mSiO2-ZnO at dosage of 200 μg per mice for 15 days.

Supplementary Table：

Table S1. Characteristics of the core-shell AuNP@mSiO_2 and AuNP@mSiO_2-ZnO from BET analysis. S_{BET} and V_t represent surface area and pore volume, respectively

Samples	S_{BET} (m^2/g)	V_t (cm^3/g)	Pore siz (nm)
**AuNP@mSiO_2**	686	0.9	2.8
**AuNP@mSiO_2-ZnO**	318	0.4	-
